# CIPK23 regulates blue light‐dependent stomatal opening in *Arabidopsis thaliana*

**DOI:** 10.1111/tpj.14955

**Published:** 2020-09-01

**Authors:** Shin‐Ichiro Inoue, Eirini Kaiserli, Xiang Zhao, Thomas Waksman, Atsushi Takemiya, Masaki Okumura, Hirotaka Takahashi, Motoaki Seki, Kazuo Shinozaki, Yaeta Endo, Tatsuya Sawasaki, Toshinori Kinoshita, Xiao Zhang, John M. Christie, Ken‐Ichiro Shimazaki

**Affiliations:** ^1^ Division of Biological Science Graduate School of Science Nagoya University Furo‐cho, Chikusa‐ku Nagoya 464‐8602 Japan; ^2^ Institute of Molecular Cell and Systems Biology College of Medical, Veterinary and Life Sciences University of Glasgow Glasgow G12 8QQ UK; ^3^ Institute of Plant Stress Biology State Key Laboratory of Cotton Biology School of Life Sciences Henan University Kaifeng 475004 People’s Republic of China; ^4^ Department of Biology Faculty of Science Kyushu University 744 Motooka Fukuoka 819‐0395 Japan; ^5^ Proteo‐Science Center Ehime University Matsuyama 790‐8577 Japan; ^6^ RIKEN Cluster for Pioneering Research 2‐1 Hirosawa Wako 351‐0198 Japan; ^7^ RIKEN Center for Sustainable Resource Science 1‐7‐22, Suehiro, Tsurumi‐ku Yokohama 230‐0045 Japan; ^8^ Gene Discovery Research Group RIKEN Center for Sustainable Resource Science 3‐1‐1 Koyadai Tsukuba 305‐0074 Japan; ^9^ Institute for the Promotion of Science and Technology Ehime University Matsuyama 790‐8577 Japan; ^10^ Institute of Transformative Bio‐Molecules (WPI‐ITbM) Nagoya University Chikusa Nagoya 464‐8602 Japan; ^11^ Present address: Department of Biology Graduate School of Sciences and Technology for Innovation Yamaguchi University Yamaguchi 753‐8512 Japan; ^12^ Present address: Department of Plant and Microbial Biology University of Minnesota

## Abstract

Phototropins (phot1 and phot2) are plant blue light receptor kinases that function to mediate phototropism, chloroplast movement, leaf flattening, and stomatal opening in Arabidopsis. Considerable progress has been made in understanding the mechanisms associated with phototropin receptor activation by light. However, the identities of phototropin signaling components are less well understood by comparison. In this study, we specifically searched for protein kinases that interact with phototropins by using an *in vitro* screening method (AlphaScreen) to profile interactions against an Arabidopsis protein kinase library. We found that CBL‐interacting protein kinase 23 (CIPK23) interacts with both phot1 and phot2. Although these interactions were verified by *in vitro* pull‐down and *in vivo* bimolecular fluorescence complementation assays, CIPK23 was not phosphorylated by phot1, as least *in vitro*. Mutants lacking CIPK23 were found to exhibit impaired stomatal opening in response to blue light but no deficits in other phototropin‐mediated responses. We further found that blue light activation of inward‐rectifying K^+^ (K^+^_in_) channels was impaired in the guard cells of *cipk23* mutants, whereas activation of the plasma membrane H^+^‐ATPase was not. The blue light activation of K^+^_in_ channels was also impaired in the mutant of BLUS1, which is one of the phototropin substrates in guard cells. We therefore conclude that CIPK23 promotes stomatal opening through activation of K^+^_in_ channels most likely in concert with BLUS1, but through a mechanism other than activation of the H^+^‐ATPase. The role of CIPK23 as a newly identified component of phototropin signaling in stomatal guard cells is discussed.

## Significance Statement

This study indicates that CIPK23 is a phototropin‐interacting protein kinase that promotes blue light‐ and phototropin‐dependent stomatal opening in *Arabidopsis thaliana*. We propose that CIPK23 does not mediate plasma membrane H^+^‐ATPase activation but mediates inward‐rectifying K^+^ channel activation in stomatal opening.

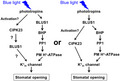

## INTRODUCTION

Phototropins (phot1 and phot2) are plasma membrane‐associated, autophosphorylating blue light receptor kinases that induce a range of physiological responses in *Arabidopsis thaliana*, which help optimize photosynthetic efficiency under weak light conditions by increasing light capture and CO_2_ absorption in leaves (Christie *et al*., 1998; Takemiya *et al*., 2005; Inoue *et al*., 2010; Gotoh *et al*., 2018). These processes include phototropism, chloroplast photorelocation movement, leaf flattening, leaf positioning, and stomatal opening (Christie, 2007; Inoue *et al*., 2008a; Demarsy and Fankhauser, 2009). Despite extensive efforts by many researchers, the primary signaling events associated with each of these responses remain largely unknown (Christie *et al*., 2015).

Many phototropin interaction partners have been identified (Inoue *et al*., 2010), four of which, ATP‐BINDING CASSETTE B19 (ABCB19), PHYTOCHROME KINASE SUBSTRATE 4 (PKS4), BLUE LIGHT SIGNALING 1 (BLUS1), and CONVERGENCE OF BLUE LIGHT AND CO2 (CBC) 1, have been shown to be direct substrate targets (Christie *et al*., 2011; Demarsy *et al*., 2012; Takemiya *et al*., 2013; Hiyama *et al*., 2017). NON‐PHOTOTROPIC HYPOCOTYL 3 (NPH3), ROOT PHOTOTROPISM 2 (RPT2), and PKS1, 2, and 4 positively regulate phototropism, leaf flattening, and leaf positioning but do not regulate chloroplast movement and stomatal opening (Motchoulski and Liscum, 1999; Sakai *et al*., 2000; Inada *et al*., 2004; Lariguet *et al*., 2006; Inoue *et al*., 2008a; de Carbonnel *et al*., 2010; Harada *et al*., 2013; Tsutsumi *et al*., 2013). While PKS4 is phosphorylated by phot1 in a blue light‐dependent manner, phosphorylation negatively regulates its action on phototropism (Demarsy *et al*., 2012; Schumacher *et al*., 2018). In addition, the auxin efflux transporter ABCB19 has been shown to be a substrate for phot1 kinase activity (Christie *et al*., 2011). Phosphorylation of ABCB19 is proposed to inhibit its efflux activity and indirectly promote auxin fluxes involved in phototropism (Christie *et al*., 2011). The guard cell‐specific protein kinase BLUS1 is phosphorylated by both phot1 and phot2 in response to blue light, and phosphorylated BLUS1 mediates stomatal opening (Takemiya *et al*., 2013). CBC1 is phosphorylated by phot1 and phot2 in guard cells, and CBC1 and the closest homolog CBC2 regulate blue light‐induced stomatal opening in both positive and negative manners (Hiyama *et al*., 2017; Hayashi *et al*., 2020). The roles of CBC1 phosphorylation are not understood at the present time. Together, these findings indicate that the primary signaling events associated with different phototropin‐mediated responses are likely to be distinct, with signal propagation diverging from the phosphorylation of specific substrates.

Blue light‐driven stomatal opening is one of the most characterized phototropin signaling pathways to date (Shimazaki *et al*., 2007; Inoue *et al*., 2010). Stomatal opening, which facilitates gaseous exchange between plants and the atmosphere, is driven by the swelling of stomatal guard cells in response to blue light (Zeiger and Hepler, 1977; Shimazaki *et al*., 2007). This swelling is achieved by increased turgor pressure in guard cells, which is induced by hyperpolarization of the plasma membrane and subsequent K^+^ uptake via inward‐rectifying K^+^ (K^+^_in_) channels at the membrane (Schroeder *et al*., 1987; Schroeder and Hedrich, 1989; Schroeder *et al*., 2001; Marten *et al*., 2010). Membrane hyperpolarization is generated by activation of the guard cell plasma membrane H^+^‐ATPase (Assmann *et al*., 1985; Shimazaki *et al*., 1986; Blatt, 1987; Yamauchi *et al*., 2016). The H^+^‐ATPase is activated in a blue light‐ and phototropin‐dependent manner by phosphorylation of its C‐terminus and subsequent binding of 14‐3‐3 proteins (Kinoshita and Shimazaki, 1999; Palmgren, 2001; Ueno *et al*., 2005; Hayashi *et al*., 2011). Phototropin autophosphorylation leads to H^+^‐ATPase activation via BLUS1 kinase activity, as well as the involvement of BLUE LIGHT‐DEPENDENT H^+^‐ATPASE PHOSPHORYLATION (BHP) and type 1 protein phosphatases (Kinoshita *et al*., 2001; Takemiya *et al*., 2006; Inoue *et al*., 2008b; Takemiya *et al*., 2013; Hayashi *et al*., 2017). However, knowledge of the signaling events coupling blue light sensing by the phototropins to activation of the H^+^‐ATPase is incomplete. For instance, the identity of the kinase responsible for phosphorylation of the H^+^‐ATPase is still lacking.

While it is well accepted that activation of the H^+^‐ATPase is the main driver of stomatal opening, there is additional evidence to support the involvement of two other modes of signaling and regulation. The first involves phototropin‐mediated suppression of plasma membrane anion channels that mediate stomatal closure, whereby their inhibition promotes stomatal opening through an increase in plasma membrane hyperpolarization (Marten *et al*., 2007). The other pathway involves phototropin modulation of K^+^_in_ channel activity, which is thought to facilitate H^+^‐ATPase‐driven stomatal opening through the uptake of K^+^ across the plasma membrane (Zhao *et al*., 2012). It is therefore possible that phototropin substrates besides BLUS1 could be involved in either of these signaling pathways. Here we adopted the Amplified Luminescent Proximity Homogeneous Assay Screen (AlphaScreen) methodology (PerkinElmer Life Sciences) to determine whether phototropins could interact with protein kinases other than BLUS1. In doing so, we identified CBL‐interacting protein kinase 23 (CIPK23) as a positive regulator in blue light‐dependent stomatal opening in Arabidopsis. Furthermore, we propose that CIPK23 constitutes a distinct signaling pathway involved in phototropin‐driven stomatal opening and contributes to this response through the activation of K^+^_in_ channels.

## RESULTS

### Identification of CIPK23 as a phototropin‐interacting protein

**Figure 1:**
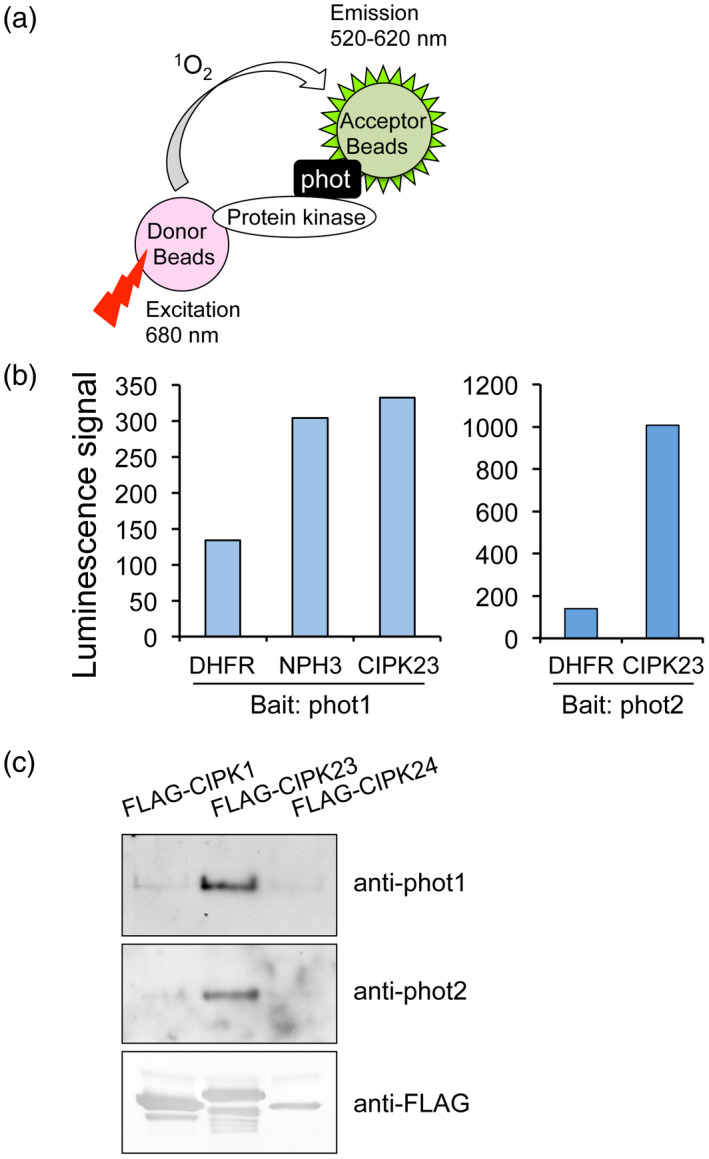
Identification of CIPK23 as an *in vitro* phototropin‐interacting protein. (a) Screening of phototropins‐interacting protein kinases using the AlphaScreen luminescence system. A protein kinase from the protein kinase library and phototropins were bound onto donor and acceptor beads, respectively. The two beads can be in close proximity only when these proteins interact. Upon excitation at 680 nm, a singlet oxygen is generated from the donor beads and transferred to the acceptor beads within 200 nm, and the resultant reaction emits light at 520–620 nm. (b) *In vitro* interaction of CIPK23 with phot1 and phot2 in the AlphaScreen method. All proteins used were expressed using a wheat germ cell‐free protein synthesis system. Interactions of phototropins with CIPK23 were detected by the AlphaScreen method as a luminescence signal. DHFR and NPH3 were used as negative and positive controls, respectively. (c) *In vitro* pull‐down assay of phototropins and CIPK23. Recombinant FLAG‐CIPK1, FLAG‐CIPK24, and FLAG‐CIPK23 were purified from *E. coli* extracts using anti‐FLAG antibody‐conjugated agarose beads, and then reacted with microsomal fractions from Arabidopsis seedlings. Co‐purified proteins were detected by immunoblotting using antibodies to phot1, phot2, and FLAG.

To identify novel kinase‐interacting partners of Arabidopsis phototropins, we performed an *in vitro* protein–protein interaction screen using AlphaScreen technology in combination with a wheat germ cell‐free protein synthesis system as reported previously (Hayashi *et al*., 2020). RIKEN Arabidopsis Full‐Length (RAFL) cDNA clones were used to construct an Arabidopsis protein kinase library (562 protein kinases) which were expressed individually by an *in vitro* transcription and translation system (Sawasaki *et al*., 2002; Sawasaki *et al*., 2004; Nemoto *et al*., 2011). In this system, a luminescent signal is generated when donor and acceptor beads are brought into close proximity. The donor beads generate singlet oxygen by excitation and the acceptor beads emit light by reacting with singlet oxygen. When two beads are in close proximity, the acceptor beads are able to receive singlet oxygen from the donor beads. The proximity of the beads depends on the interaction between proteins that have been conjugated onto the beads (Figure 1a). Phot1 or phot2 and each protein kinase from the library were conjugated on acceptor and donor beads as bait and prey, respectively. We then screened this library for kinases that interact with phot1 and phot2 using the AlphaScreen approach and identified CIPK23 as a candidate. A negative control protein, dihydrofolate reductase (DHFR), showed a slight interaction with phot1 and phot2 in this system that was expressed as a luminescence signal in the AlphaScreen (Figure 1b). By contrast, CIPK23 showed a high level of interaction with both phot1 and phot2. The extent of CIPK23 binding to phot1 was similar to that of NPH3 to phot1, which served as a positive control in our analysis (Motchoulski and Liscum, 1999; de Carbonnel *et al*., 2010).

The interaction between phototropins and CIPK23 was confirmed by *in vitro* pull‐down assay. FLAG‐tagged CIPK1, CIPK23, or CIPK24 was expressed and purified from *Escherichia coli* cells using anti‐FLAG antibody‐conjugated agarose beads. Purified FLAG‐tagged CIPK proteins were then incubated with microsomal membranes from Arabidopsis seedlings. Immunoprecipitation analysis showed that FLAG‐CIPK23 co‐purified with both phot1 and phot2 from microsomal membrane fractions, but CIPK1 and CIPK24 did not (Figure 1c). These results indicate that CIPK23 interacts with both phot1 and phot2 *in vitro*.

**Figure 2:**
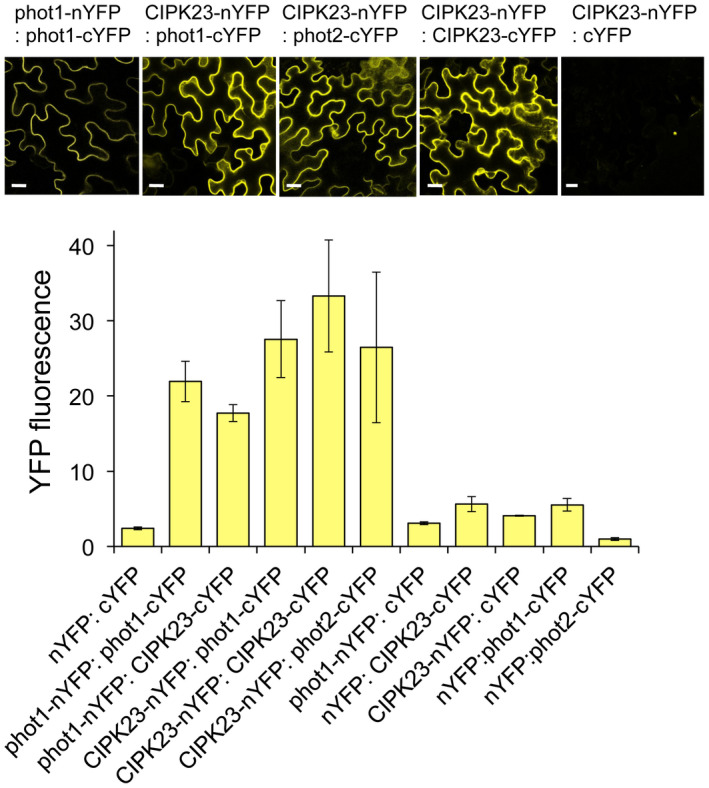
BiFC analysis showing the interactions between phototropins and CIPK23 and CIPK23 homodimerization in *Nicotiana benthamiana* leaf epidermal cells. Indicated fusion proteins were transiently co‐expressed in *Nicotiana* cells via *Agrobacterium*. nYFP and cYFP represent N‐ and C‐terminal halves of the YFP protein, respectively. Phot1 homodimerization was used as a positive control. Representative confocal images show reconstitution of YFP fluorescence upon interaction. Representative images of negative controls are shown in Figure S1. Quantitative measurements of the interaction are plotted based on total YFP fluorescence in test and control infiltrations (*n* = 7). Scale bars = 20 μm.

To verify the occurrence of the CIPK23–phototropin interactions in plant cells, we performed bimolecular fluorescence complementation (BiFC) in tobacco (*Nicotiana benthamiana*) leaves as reported previously (Kaiserli *et al*., 2009). Both phot1 and phot2 were found to interact with CIPK23 in the tobacco epidermal cells (Figure 2), whereas no BiFC signal was detected when CIPK24 and empty vectors were used in the series of co‐expression experiments (Figure 2; Figures S1 and S2). Taken together, these findings further demonstrate that CIPK23 specifically interacts with phot1 and phot2 *in vivo*.

### CIPK23 is not required for phototropin‐mediated phototropism, chloroplast movement, leaf flattening, and promotion of plant growth

To investigate the functions of CIPK23 in phototropin‐mediated blue light responses, we obtained two transfer DNA insertional knockout mutants, *cipk23‐1* (SALK_032341) and *cipk23‐5* (SALK_138057) (Figure S3a; Cheong *et al*., 2007; Nieves‐Cordones *et al*., 2012). We confirmed the absence of transcripts of full‐length *CIPK23* in either of these knockout mutants by reverse transcriptase (RT)‐PCR (Figure S3b).

We next confirmed the abundance of *CIPK23* transcripts in various tissues from 4‐week‐old Arabidopsis plants. RT‐PCR analysis indicated that *CIPK23* transcripts were expressed in guard cell protoplasts (GCPs), mesophyll cell protoplasts (MCPs), rosette leaves, petioles, inflorescence stems, and roots with a higher level being detected in GCPs (Figure S3c). Ubiquitous expression of *CIPK23* was previously shown by promoter‐GUS expression assay (Cheong *et al*., 2007) and ubiquitous expression of phot1 and phot2 proteins was similarly confirmed (Kagawa *et al*., 2001; Sakamoto and Briggs, 2002). These findings suggest that CIPK23 could play a role in phototropin‐mediated responses.

**Figure 3:**
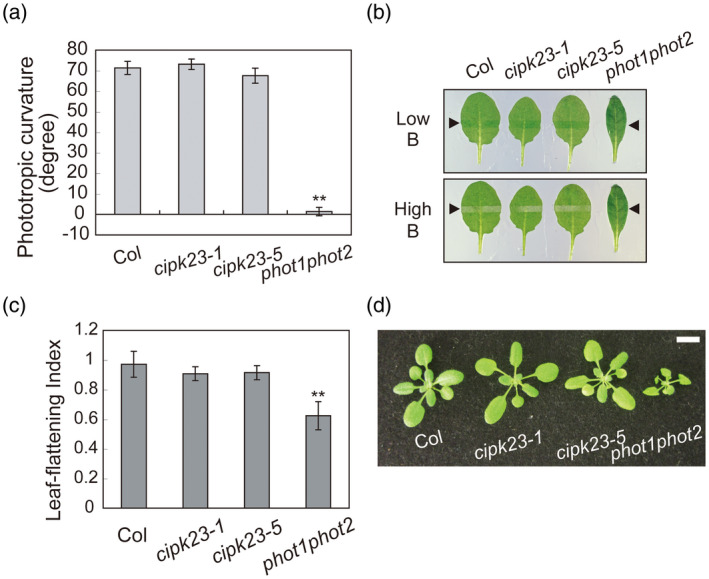
Effects of *CIPK23* mutations on various phototropin‐mediated blue light responses. (a) Phototropism of *Arabidopsis thaliana* wild‐type (Col), *cipk23‐1*, *cipk23‐5*, and *phot1phot2*. Etiolated seedlings were irradiated with unilateral blue light at 0.1 µmol m^−2^ sec^−1^ for 14 h. Values are means ± SE (*n* = 30–54). Differences from wild‐type plants were evaluated using Student’s *t* test (^**^ *P* < 0.01). (b) Chloroplast photorelocation movement in wild‐type (Col), *cipk23‐1*, *cipk23‐5*, and *phot1phot2* leaves. Slit band assays were performed to observe chloroplast movement. Rosette leaves were detached and irradiated with blue light for 30 min through a 1‐mm slit. Blue light was irradiated at 1 µmol m^−2^ sec^−1^ to evaluate the accumulation response, and at 90 µmol m^−2^ sec^−1^ for the avoidance response. Upper and lower panels indicate chloroplast accumulation and avoidance responses, respectively. Black arrowheads indicate irradiated areas. (c) Leaf flattening of wild‐type (Col), *cipk23‐1*, *cipk23‐5*, and *phot1phot2* leaves. Plants were grown on soil under fluorescent light (50 µmol m^−2^ sec^−1^) for 5 weeks. The leaf‐flattening index was expressed as the ratio of projection of the leaf before and after artificial uncurling. Values are means ± SE (*n* = 5). Differences from wild‐type were evaluated using Student’s *t* test (^**^ *P* < 0.01). (d) Growth and morphology of wild‐type (Col), *cipk23‐1*, *cipk23‐5*, and *phot1phot2*. Plants were grown on soil for 4 weeks and photographed. The bar indicates 1 cm.

We explored whether phototropin‐mediated responses were altered in *cipk23* mutants given that CIPK23 is ubiquitously expressed. First, phototropic curvature was determined using etiolated seedlings. The hypocotyls of *cipk23‐1* and *cipk23‐5* mutants showed phototropic bending in response to unilateral blue light (0.1 µmol m^−2^ sec^−1^) comparable to that found for wild‐type (Col) seedlings, unlike the *phot1phot2* double mutant (Figure 3a). Next, we determined whether blue light‐induced chloroplast relocation movements were altered in *cipk23* mutants by slit band assay (Suetsugu *et al*., 2005; Inoue *et al*., 2011) (Figure 3b). Irradiation of wild‐type leaves with weak blue light (1 µmol m^−2^ sec^−1^) through a 1‐mm slit promotes chloroplast accumulation in this region and the appearance of a darker green band (Figure 3b: upper panel). Conversely, a white band is produced when this area is irradiated with strong blue light (90 µmol m^−2^ sec^−1^) owing to chloroplast avoidance movement (Figure 3b: lower panel). Similarly, leaves of *cipk23* mutants showed both green and white bands in response to weak and strong blue light, respectively. These results indicate that *cipk23* mutants were not altered in chloroplast accumulation and avoidance responses.

The rosette leaves of the *phot1phot2* double mutant are epinastic and curl downward at the side in comparison to the leaves from wild‐type plants (Figure 3c). This leaf‐flattening response was still apparent in *cipk23* mutants. Moreover, *cipk23* mutants showed normal growth under our conditions, comparable to that of wild‐type plants, unlike the *phot1phot2* double mutant (Figure 3d).

Taken together, the above findings demonstrate that mutants lacking CIPK23 are not altered in phototropin‐mediated phototropism, chloroplast movement, and leaf flattening in the tested conditions.

### CIPK23 is required for phototropin‐mediated stomatal opening

**Figure 4:**
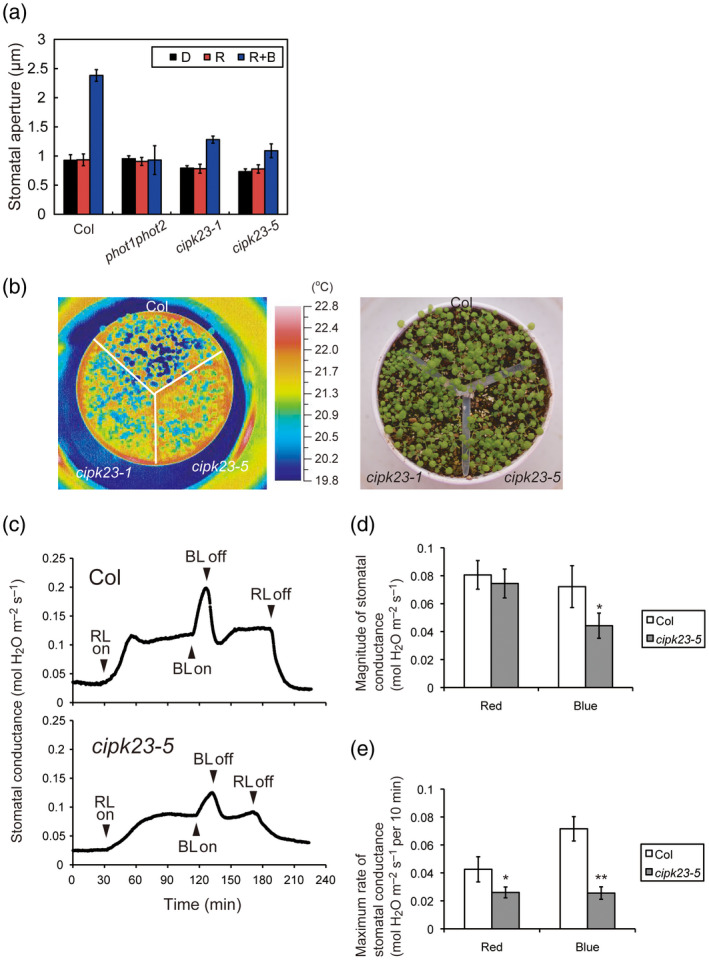
Effect of *CIPK23* mutations on blue light‐dependent stomatal opening. (a) Blue light‐dependent stomatal opening in the epidermis of wild‐type (Col), *phot1phot2*, *cipk23‐1*, and *cipk23‐5* plants. Epidermal peels were isolated from dark‐adapted plants, and irradiated with red light (50 µmol m^−2^ sec^−1^; R) with or without blue light (10 µmol m^−2^ sec^−1^; R + B) for 3 h. Values are means of three independent experiments with standard deviations. In each experiment, 45 stomata were measured. (b) Infrared thermal images of wild‐type (Col), *cipk23‐1*, and *cipk23‐5* plants. Plants were grown in well‐watered conditions for 3 weeks under white light at 50 µmol m^−2^ sec^−1^. (c) Stomatal conductance changes in response to red and blue light in intact leaves from wild‐type (Col) and *cipk23‐5* plants. Red light (600 µmol m^−2^ sec^−1^; RL) and blue light (10 µmol m^−2^ sec^−1^; BL) were switched on/off at the times indicated by the arrowheads. (d, e) Magnitude (d) and the maximum rate (e) of stomatal conductance in response to red and blue light. Data represent the means ± SD of four independent experiments. Differences were evaluated using Student’s *t* test (*P < 0.05, **P < 0.01).

Given the prevalence of *CIPK23* expression in GCPs (Figure S3c), we determined whether blue light‐dependent stomatal opening was altered in the *cipk23* mutants. Stomata in the leaf epidermis of wild‐type plants opened in response to blue light under a background of red light, but did not open in response to red light treatment (Figure 4a). This blue light response is mediated by the phototropins and is absent in the stomata of the *phot1phot2* double mutant (Inoue *et al*., 2008b; Inoue *et al*., 2011; Hayashi *et al*., 2017). Blue light‐dependent stomatal opening was found to be impaired in epidermal peels isolated from *cipk23‐1* and *cipk23‐5* mutants (Figure 4a).

The defect in blue light‐induced stomatal opening observed in *cipk23* mutants was investigated in more detail. Changes in leaf temperature are known to reflect differences in stomatal aperture. For instance, water evaporation via open stomata results in a decrease in leaf temperature (Merlot *et al*., 2002; Hashimoto *et al*., 2006; Takemiya *et al*., 2013; Inoue *et al*., 2017). The leaf temperature of *cipk23* mutants was higher compared to that of wild‐type leaves under our growth conditions (Figure 4b), consistent with their inability to open stomata in response to blue light (Figure 4a). The result suggests that water evaporation is reduced in *cipk23* mutants since stomatal opening is reduced under the light conditions examined.

Finally, we measured the stomatal conductance of intact leaves in response to light (Figure 4c). Stomatal conductance in wild‐type leaves increased in response to strong red light (600 µmol m^−2^ sec^−1^) and reached a steady state after approximately 30 min (Figure 4c: upper graph). By comparison, this rate of increase was slightly reduced in the *cipk23‐5* mutant (Figure 4c,e). Stomatal conductance in wild‐type leaves was further increased following irradiation with weak blue light (10 µmol m^−2^ sec^−1^) superimposed onto the background of red light (Figure 4c: upper graph). However, the magnitude and rate of this increase was largely reduced in the leaves of the *cipk23‐5* mutant (Figure 4c–e). From the analysis of stomatal responses, we conclude that CIPK23 plays a role in mediating blue light‐dependent stomatal opening in Arabidopsis.

### Blue light activation of the plasma membrane H^+^‐ATPase is unaltered in the guard cells of *cipk23* mutants

Blue light activates the plasma membrane H^+^‐ATPase via phototropins through phosphorylation in guard cells. Activation of the H^+^‐ATPase generates the driving force for stomatal opening (Shimazaki *et al*., 2007; Inoue *et al*., 2010; Yamauchi *et al*., 2016; Inoue and Kinoshita, 2017). We therefore prepared GCPs from the rosette leaves of wild‐type and *cipk23‐5* mutant plants, and measured H^+^ pumping activity in response to blue light (Figure S4a). GCPs from wild‐type or *cipk23‐5* plants both showed a similar magnitude and rate of H^+^ pumping in response to blue light (Figure S4a; Table S1). We further determined whether blue light‐dependent phosphorylation of the H^+^‐ATPase was altered in GCPs isolated from *cipk23‐5* through 14‐3‐3 protein binding by far‐Western blotting (Figure S4b). GCPs from the *cipk23‐5* mutant exhibited similar levels of H^+^‐ATPase phosphorylation to those detected in GCPs from wild‐type rosette leaves. Moreover, the abundance of H^+^‐ATPase was unaltered in GCPs from the *cipk23‐5* mutant. These results indicate that the H^+^‐ATPase is normally activated by blue light in the absence of CIPK23 and further suggest that signaling components downstream of the H^+^‐ATPase were impaired in *cipk23* mutants.

**Figure 5:**
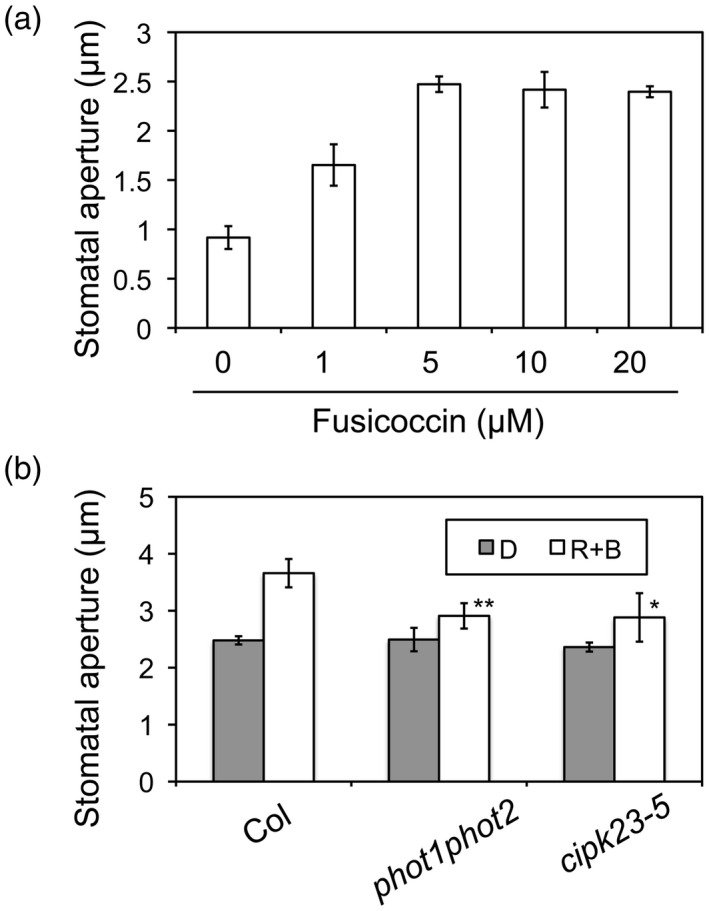
Effect of blue light on fusicoccin (FC)‐induced stomatal opening. (a) Stomatal opening in response to FC in the epidermis of wild‐type (Col) leaves. Epidermal peels from dark‐adapted plants were incubated with FC at the indicated concentrations for 3 h in darkness. Data represent the means ± SD of three independent experiments. In each experiment, 45 stomata were measured. (b) Effect of blue light on FC‐induced stomatal opening. Epidermal peels were incubated with FC at 10 µm with or without mixed light (R + B: red light at 50 µmol m^−2^ sec^−1^ and blue light at 10 µmol m^−2^ sec^−1^) for 3 h. Differences were evaluated using Student’s *t* test (*P < 0.05, **P < 0.01).

The fungal toxin fusicoccin (FC) directly activates the plasma membrane H^+^‐ATPase and induces stomatal opening in the dark (Kinoshita and Shimazaki, 2001; Kinoshita *et al*., 2001). In darkness, the stomata in the epidermis of wild‐type Arabidopsis opened in response to FC treatment in a dose‐dependent manner and the aperture reached a maximum at 5 µm (Figure 5a). Stomata of the *cipk23‐1* and *cipk23‐5* mutants showed a similar response to 10 µm FC treatment as did those from wild‐type plants, as well as the *phot1phot2* double mutant in the dark (Figure S5). Interestingly, further increases in stomatal opening were observed in the epidermis of wild‐type plants when blue light was introduced following FC treatment (Figure 5b). By contrast, stomata in the *phot1phot2* double mutant did not show this increase in stomatal opening after blue light irradiation. These data therefore provide evidence for the presence of other factors in addition to the H^+^‐ATPase that are required for phototropin‐mediated stomatal opening. These likely include CIPK23 since the *cipk23‐5* mutant also failed to show an increase in stomatal opening when irradiated with blue light following FC treatment in darkness (Figure 5b).

### CIPK23 is required for blue light activation of K^+^_in_ channels

**Figure 6:**
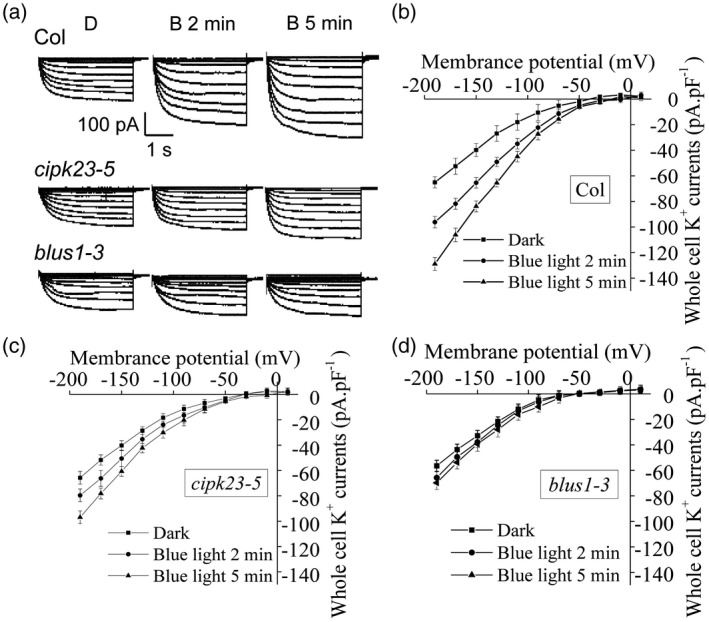
Effect of *CIPK23* and *BLUS1* mutations on blue light‐induced activation of K^+^_in_ currents in guard cell protoplasts. (a) Effect of blue light on voltage‐dependent K^+^_in_ currents in GCPs from wild‐type (Col), *cipk23‐5*, and *blus1‐3* plants. Whole‐cell K^+^_in_ currents (pA) were measured 2 and 5 min after the pulse of blue light (100 μmol m^−2^ sec^−1^, 30 sec). Measurements were performed under dim red light (0.2 μmol m^−2^ sec^−1^) as a control. (b–d) The relationship between the whole‐cell inward‐rectifying K^+^ currents (pA pAF^−1^) and the membrane potential (mV) in GCPs from wild‐type (Col) (b), *cipk23‐5* (c), and *blus1‐3* (d) plants. Each value is the mean current from six or eight independent experiments, and the error bars denote SE.

Recent experiments, using a voltage‐clamp technique, have demonstrated that blue light increased plasma membrane K^+^_in_ channel currents by about 50% in guard cells and this increment was completely lost in *phot1phot2* guard cells (Zhao *et al*., 2012). In addition, CIPK23 promotes K^+^ uptake through direct activation of the AKT1 K^+^_in_ channel in roots (Li *et al*., 2006; Xu *et al*., 2006). Given our present findings, we rationalized that CIPK23 could be involved in mediating the signaling between the phototropins and K^+^_in_ channels. To test this possibility, we determined the activity of K^+^_in_ channels in GCPs isolated from the *cipk23‐5* mutant by monitoring the K^+^ current by whole‐cell patch clamping. No difference in K^+^_in_ channel activity was observed between wild‐type GCPs and those from the *cipk23‐5* mutant under dark conditions (Figure 6a). A short pulse (30 sec) of blue light led to a rapid (within 5 min), 2‐fold increase in K^+^_in_ channel activity in wild‐type GCPs, as reported previously (Figure 6a; Zhao *et al*., 2012). By contrast, this change in K^+^ current in response to blue light was highly diminished in GCPs from the *cipk23‐5* mutant (Figure 6a). Steady‐state current–voltage curves also showed that K^+^_in_ currents increased in response to blue light in wild‐type GCPs, whereas this was decreased in the case of *cipk23‐5* (Figure 6b,c; Figure S6a,b). These findings therefore suggest that blue light‐induced changes in guard cell K^+^_in_ currents are impaired in the *cipk23‐5* mutant. Thus, CIPK23 likely couples phototropin activation to changes in K^+^_in_ currents at the guard cell plasma membrane.

Recent studies have shown that the guard cell‐specific kinase BLUS1, which mediates blue light‐dependent stomatal opening, acts as a substrate for phototropin kinase activity and forms a complex with phot1 and phot2 (Takemiya *et al*., 2013; Schnabel *et al*., 2018). Thus, similar electrophysiological experiments were performed using GCPs from the *blus1‐3* mutant. Blue light‐induced changes in guard cell K^+^_in_ currents were found to be impaired in the guard cells of *blus1‐3* (Figure 6a,b,d; Figure S6a,c). We therefore conclude that BLUS1 also participates in the signaling for the K^+^_in_ channel activation by blue light.

### We have no evidence that phototropin kinases phosphorylate CIPK23

Phototropins may directly phosphorylate CIPK23 in response to blue light since we found that CIPK23 interacts with both phot1 and phot2 (Figures 1 and 2). To determine whether or not CIPK23 is a direct substrate for phototropin kinase activity, we performed *in vitro* thio‐phosphorylation assays using the gatekeeper‐engineered variant of phot1, phot1_T740G_, and *N^6^*‐benzyl‐ATPγS as a phosphodonor, as reported recently (Schnabel *et al*., 2018). Blue light‐dependent autophosphorylation of phot1 was clearly observed as a consequence of thio‐phosphorylation (Figure S7: white arrowhead). By contrast, a thio‐phosphorylation signal corresponding to CIPK23 phosphorylation by phot1 was not observed (Figure S7: black arrowhead). From these experiments, we conclude that CIPK23 is not a substrate for phototropin kinase, at least in the gatekeeper/thio‐phosphorylation system.

## DISCUSSION

### CIPK23 is a positive regulator of phototropin‐mediated stomatal opening

Mutants lacking CIPK23 showed impaired blue light‐dependent stomatal opening in both experiments using epidermal fragments and intact leaves (Figure 4). FC‐induced stomatal opening in darkness was unaffected in the *cipk23‐5* mutant but was affected under blue light conditions (Figure S5; Figure 5b). Furthermore, the plasma membrane H^+^‐ATPase was normally activated in response to blue light in *cipk23‐5* guard cells (Figure S4). Thus, we assume that CIPK23 is involved in a signaling pathway which is different from that associated with phototropin‐dependent activation of the plasma membrane H^+^‐ATPase for stomatal opening. Finally, we found that blue light activation of the K^+^_in_ channel current was impaired in the guard cells of the *cipk23‐5* mutant compared to wild‐type guard cells (Figure 6; Figure S6). From these results, we conclude that CIPK23 acts as a positive regulator in stomatal opening and mediates blue light signaling from phototropins to the K^+^_in_ channels in guard cells (Figure S10). However, previous studies have indicated that CIPK23 functions as a negative regulator of ABA or water stress signaling in guard cells (Cheong *et al*., 2007; Nieves‐Cordones *et al*., 2012). In this case, mutations in *CIPK23* were found to enhance ABA‐ or water stress‐dependent inhibition of light‐induced stomatal opening. It is therefore reasonable to interpret that CIPK23 acts as a positive regulator of phototropin‐mediated stomatal opening through the activation of K^+^_in_ channels, and this action appears to antagonize ABA‐induced stomatal closure.

CIPK23 is a member of the SnRK3 subfamily that mainly acts in adaptive responses through the regulation of ion transporters (Hrabak *et al*., 2003; Luan, 2008; Sanyal *et al*., 2015). CIPK23 stimulates K^+^ and nitrate uptake through phosphorylation‐dependent activation of the high‐affinity K^+^ transporter 5 (HAK5), the K^+^_in_ channel AKT1, and the nitrate transporter CHL1/NRT1.1 in the plasma membrane of roots under low K^+^ and N conditions (Li *et al*., 2006; Xu *et al*., 2006; Ho *et al*., 2009; Ragel *et al*., 2015). Since uptake of K^+^ and nitrate into guard cells contributes to stomatal opening (Schroeder *et al*., 2001; Guo *et al*., 2003; Shimazaki *et al*., 2007), these studies provide further support that CIPK23 acts as a positive regulator of stomatal opening through the activation of ion transporters downstream of the phototropins. The work presented here highlights an additional level of complexity associated with phototropin signaling events required for transporter regulation and stomatal opening.

A recent electrophysiological study using transient expression in *Xenopus* oocytes suggested that CIPK23 potentially functions as a positive regulator in ABA‐induced stomatal closure through activation of the S‐type anion channel SLAC1 (Maierhofer *et al*., 2014). There is a conclusion discrepancy between Maierhofer *et al*. (2014) and this study. However, *slac1* mutants show large stomatal opening and impairment of ABA‐induced stomatal closure (Negi *et al*., 2008; Vahisalu *et al*., 2008). In contrast, *cipk23* mutants did not show such open stomata phenotypes but showed closed stomata phenotypes (Cheong *et al*., 2007; Nieves‐Cordones *et al*., 2012; Figure 4). From these findings, we conclude that SLAC1 activation by CIPK23 may not strongly contribute to regulation of stomatal aperture in our experimental conditions. Further electrophysiological measurements of anion channel activity in *cipk23* guard cells are therefore needed to clarify how CIPK23 regulates SLAC1 under our experimental conditions.

### Regulation of K^+^_in_ channel activity by CIPK23 in guard cells

It is well established that blue light activates the guard cell H^+^‐ATPase via the phototropins, resulting in hyperpolarization of the plasma membrane, which in turn activates voltage‐dependent K^+^_in_ channels that are required for K^+^ uptake (Schroeder *et al*., 2001; Shimazaki *et al*., 2007; Inoue *et al*., 2010). However, in addition to this single‐scheme signaling, recent findings have demonstrated that blue light also enhances the K^+^_in_ channel current in guard cells via phototropins (Zhao *et al*., 2012). Since this enhancement is observed under the membrane voltage‐clamped conditions, it is thought that the effect of blue light on the K^+^_in_ channel current is independent from plasma membrane H^+^‐ATPase activity. In the present study, we found that *cipk23‐5* exhibited impaired stomatal opening and lacked K^+^_in_ channel activation in response to blue light (Figures 4, 5, 6; Figure S6). In addition, activation and phosphorylation of the H^+^‐ATPase in response to blue light were not affected in *cipk23‐5* guard cells (Figure S4). From these results, we conclude that CIPK23 does not mediate signaling from the phototropins to H^+^‐ATPase activation but activates K^+^_in_ channels via the phototropins (Figure S10). Blue light‐dependent stomatal opening is completely abolished in the *phot1phot2* mutant but not completely in *cipk23* mutants (Figure 4), suggesting that the blue light activation of K^+^_in_ channels partially contributes to stomatal opening and phototropin‐mediated activation of both the H^+^‐ATPase and K^+^_in_ channels is needed for full stomatal opening.

AKT1 is a guard cell‐expressing K^+^_in_ channel that functions in stomatal opening together with other K^+^_in_ channels, including KAT1, KAT2, and AKT2 (Véry and Sentenac, 2003; Gambale and Uozumi, 2006; Harada and Shimazaki, 2009; Takahashi *et al*., 2013). The *kincless* mutant was generated by expressing a dominant‐negative variant of *KAT2* in the *kat2* mutant background. Guard cell K^+^_in_ currents are lost in the *kincless* mutant (Lebaudy *et al*., 2008), which shows a strong impairment in blue light‐dependent stomatal opening (Takahashi *et al*., 2013). CIPK23 has been shown to activate AKT1 by direct phosphorylation to promote K^+^ uptake in roots under low K^+^ conditions (Li *et al*., 2006; Xu *et al*., 2006; Sanchez‐Barrena *et al*., 2020). On the basis of these findings, we propose that CIPK23 could also activate guard cell AKT1 by direct protein phosphorylation in response to blue light. Indeed, CIPK23 has been shown to specifically interact with AKT1 among other plant K^+^_in_ channels in yeast (Li *et al*., 2006). Alternatively, CIPK23 could phosphorylate and regulate the activity of other K^+^_in_ channels in guard cells either directly or indirectly via scaffold proteins. Further investigation will therefore be required to clarify the role of CIPK23 in regulating K^+^_in_ channel activity in phototropin signaling.

The CIPK protein family consists of 26 members in Arabidopsis. Phylogenetic tree analysis indicates that CIPK23 is localized in the clade containing CIPK3, CIPK9, and CIPK26 (Figure S8). According to a public microarray database eFP browser (http://bar.utoronto.ca/efp/cgi‐bin/efpWeb.cgi?dataSource=Guard_Cell), *CIPK3* and *CIPK9* are expressed in guard cells at similar levels to *CIPK23* (Figure S9). These expression data suggest that CIPK3, CIPK9, and CIPK26 may function redundantly with CIPK23 in the regulation of stomatal opening. However, Luan’s group has demonstrated that members outside this clade, namely, CIPK6 and CIPK16, have similar functions to CIPK23 with respect to AKT1 activation (Lee *et al*., 2007). Expression of *CIPK6* is also apparent in guard cells (Figure S9), suggesting that it may also have a similar function to CIPK23 in stomatal opening.

We found that blue light increased K^+^_in_ channel currents 2‐fold in wild‐type guard cells (Zhao *et al*., 2012; Figure 6a,b), and this enhancement in K^+^_in_ channel activity appears to contribute to stomatal opening. However, it has already been reported that *Arabidopsis* guard cells possess sufficient K^+^_in_ channel activity required for stomatal opening (Szyroki *et al*., 2001; Lebaudy *et al*., 2008; Takahashi *et al*., 2013). Stomatal opening is affected only when K^+^_in_ channel activity is largely reduced (by 70–80%). It is therefore difficult to attribute the impairment in stomatal opening in *cipk23* mutants solely to a failure in K^+^_in_ channel activation. Other ion transporters, such as nitrate transporters activated through CIPK23 (Guo *et al*., 2003; Ho *et al*., 2009), could also play an important role in stomatal opening in response to blue light.

### Activation of CIPK23 via the phototropins

One question arising from our findings is how the phototropins activate CIPK23 in response to blue light. Structural and biochemical analyses suggest that CIPK23 is activated by binding of the calcium sensor calcineurin B‐like (CBL) 1/9 in a Ca^2+^‐dependent manner (Luan, 2008) and/or phosphorylation by another protein kinase (Chaves‐Sanjuan *et al*., 2014). The activation of CIPK23 in guard cells may therefore require an increase in cytosolic Ca^2+^ or phosphorylation of CIPK23 by an upstream protein kinase. At least for the former, previous reports have demonstrated that cytosolic Ca^2+^ increases in response to ABA or high concentrations of CO_2_ in guard cells contribute to stomatal closure (McAinsh *et al*., 1990; Schroeder and Hagiwara, 1990; Allen *et al*., 1999, 2001; Kim *et al*., 2010). Indeed, guard cell K^+^_in_ channel currents have shown to be blocked by cytosolic Ca^2+^ increases in electrophysiological experiments (Grabov and Blatt, 1998, 1999; Wang *et al*., 2013). In addition, the plasma membrane‐anchoring of the CIPK23 kinase domain strongly increased K^+^_in_ channel currents in the absence of CBL interactions (Lee *et al*., 2007). These observations suggest that CIPK23 activity for stomatal opening may be independent from cytosolic Ca^2+^ increases and the function of CBL1/9 may be required for the recruitment of CIPK23 to the plasma membrane.

A guard cell‐specific kinase BLUS1, which acts as a substrate for phototropins, mediates blue light‐dependent stomatal opening through H^+^‐ATPase activation (Takemiya *et al*., 2013). In addition, our electrophysiological experiments indicated that blue light‐dependent activation of K^+^_in_ channel currents was severely impaired in the *blus1‐3* guard cells (Figure 6a,d; Figure S6a,c). These results suggest that BLUS1 activates both the plasma membrane H^+^‐ATPase and the K^+^_in_ channel, but CIPK23 only activates the K^+^_in_ channel in stomatal opening. On the basis of these findings, we speculate that CIPK23 may regulate BLUS1 in blue light activation of the K^+^_in_ channels (Figure S10: left model). In the present study, phot1 did not phosphorylate CIPK23 *in vitro* (Figure S7), although these proteins were shown to physically interact *in vitro* and *in vivo* (Figures 1 and 2). Since CIPK23 is likely to form a protein complex with phototropins and BLUS1, another potential signaling scenario could involve CIPK23 acting as a substrate for BLUS1 (Figure S10: right model). However, we have not been successful to date in determining whether or not CIPK23 is a substrate for BLUS1 kinase activity because of the difficulty in producing active recombinant BLUS1 (Hayashi *et al*., 2017). Further experiments are now needed to clarify these points and improve our understanding of how CIPK23 regulates phototropin signaling.

## EXPERIMENTAL PROCEDURES

### Plant materials and growth conditions

*Arabidopsis thaliana* and *N. benthamiana* were grown on soil with a 16‐h light/8‐h dark photoperiod under white fluorescent light (50 µmol m^−2^ sec^−1^) at 22–24°C. Four‐ to five‐week‐old plants were used for stomatal bioassays, isolation of GCPs, and preparation of total RNA.

We obtained *cipk23‐1* (SALK_032341) and *cipk23‐5* (SALK_138057) from the Arabidopsis Biological Resource Center and isolated homozygous mutants by PCR using genomic DNA according to the SIGnAL website (http://signal.salk.edu). We used both mutants for phenotypic analyses after confirmation of knockout mutants by RT‐PCR (Figure S3b).

### Protein kinases screening by using the AlphaScreen

All proteins for the AlphaScreen method were expressed using a wheat germ cell‐free protein synthesis system (Sawasaki *et al*., 2002, 2004, 2005). Full‐length cDNAs of *PHOT1* and *PHOT2* were amplified by RT‐PCR from the wild‐type (Col) cDNAs using the following oligonucleotide primers: 5′‐CCCAAGCTTATGGAACCAACAGAAAAACCATCG‐3′ and 5′‐CCCAAGCTTTCAAAAAACATTTGTTTGCAGATCTTC‐3′ for *PHOT1* and 5′‐ATGGAGAGGCCAAGAGCCCCTCC‐3′ and 5′‐TTAGAAGAGGTCAATGTCCAAGTCC‐3′ for *PHOT2*. The amplified *PHOT1* and *PHOT2* fragments were cloned into the pFLAG‐MAC vector (Sigma‐Aldrich) via *Hind*III and *Sma*I sites, respectively. The coding regions of FLAG‐tagged phototropins were amplified by PCR from these vectors using the following primers: 5′‐GGGGTACCATGGACTACAAGGACGACGATGAC‐3′ and 5′‐GGGGTACCTCAAAAAACATTTGTTTGCAGATCTTC‐3′ for *FLAG*‐*PHOT1* and 5′‐GGGGTACCATGGACTACAAGGACGACGATGAC‐3′ and 5′‐GGGGTACCTTAGAAGAGGTCAATGTCCAAGTCC‐3′ for *FLAG*‐*PHOT2*. The DNA fragments were cloned into the *Kpn*I site of the pEU3‐NII vector, and resulting plasmids were used for the protein synthesis of FLAG‐phot1 and FLAG‐phot2 as baits for the protein–protein interaction screening. Each of the full‐length cDNAs of Arabidopsis protein kinases (562 members) was amplified from the RAFL cDNA library and added to the DNA regions of the SP6 promoter and N‐terminal biotin ligation site by split‐primer PCR as described previously (Sawasaki *et al*., 2002, 2004; Nemoto *et al*., 2011). *In vitro* transcription, translation, and biotin labeling were performed according to previous methods (Sawasaki *et al*., 2005; Takahashi *et al*., 2009; Nemoto *et al*., 2011). FLAG‐tagged phot1 and phot2 were synthesized in the presence of 100 µm flavin mononucleotide as a chromophore (Sawasaki *et al*., 2004).

To detect the interactions between FLAG‐tagged phototropins and protein kinases, 5 µl of each protein‐synthesized mixture was mixed and reacted in a 20 µl reaction mixture containing 50 mm Tris‐HCl (pH 7.6), 100 mm potassium acetate, 10 mm MgCl_2_, 0.1 mm DTT, 5 µg ml^−1^ anti‐FLAG antibody (Sigma‐Aldrich), 1 mg ml^−1^ BSA, 0.1 µl streptavidin‐coated donor beads, and 0.1 µl anti‐IgG acceptor beads at 23°C for 1 h. Luminescent signals were analyzed by the AlphaScreen detection program (PerkinElmer Life and Analytical Sciences; Takahashi *et al*., 2009).

### *In vitro* pull‐down assays

Pull‐down assays were carried out using recombinant CIPKs expressed and purified from *E. coli* in combination with microsomal membranes from Arabidopsis seedlings. Full‐length cDNAs of *CIPK1*, *CIPK23*, and *CIPK24* were amplified by RT‐PCR using the following oligonucleotide primers: 5′‐CCCAAGCTTATGGTGAGAAGGCAAGAGGAGG‐3′ and 5′‐CCCAAGCTTCTAAGTTACTATCTCTTGCTCCGGCG‐3′ for CIPK1, 5′‐CCCAAGCTTATGGCTTCTCGAACAACGCCTTCAC‐3′ and 5′‐CCCAAGCTTTTATGTCGACTGTTTTGCAATTGTCCG‐3′ for CIPK23, and 5′‐CCCAAGCTTATGACAAAGAAAATGAGAAGAGTGGGC‐3′ and 5′‐CCCAAGCTTTCAAAACGTGATTGTTCTGAGAATCTC‐3′ for CIPK24. The amplified DNA fragment was cloned into the *Hin*dIII site of the pFLAG‐MAC Expression Vector (Sigma‐Aldrich). The resulting plasmid vectors were transformed into the *E. coli* BL21 strain. The recombinant CIPK proteins were expressed as a fusion protein with FLAG‐tag and purified from *E. coli* extracts using anti‐FLAG agarose (Sigma‐Aldrich) according to the manufacturer’s instructions (https://www.sigmaaldrich.com/content/dam/sigma‐aldrich/docs/Sigma/Bulletin/f2426bul.pdf). The agarose beads were then incubated with microsomal proteins (100 µg) for 2 h at 4°C. Proteins were solubilized from the beads by adding a half volume of SDS sample buffer containing 4.5% SDS, 30% sucrose, 22.5% β‐mercaptoethanol, 0.018% Coomassie Brilliant Blue, 4.5 mm EDTA, and 45 mm Tris‐HCl (pH 8.0) and subjected to SDS‐PAGE. Proteins were immunodetected by using anti‐phot1, anti‐phot2, and anti‐FLAG monoclonal antibodies (Sigma‐Aldrich) according to a previously described method (Inoue *et al*., 2011).

### BiFC assays

Full‐length cDNAs of *CIPK23*, *CIPK24*, and *PHOT2* were amplified by RT‐PCR using the following oligonucleotide primers: 5′‐GCCTCTAGAATGGCTTCTCGAACAACGCCTTCAC‐3′ and 5′‐ATCCCGGGTGTCGACTGTTTTGCAATTGTCCG‐3′ for *CIPK23*, 5′‐GCCTCTAGAATGACAAAGAAAATGAGAAGAGTGGGC‐3′ and 5′‐ATCCCGGGAAACGTGATTGTTCTGAGAATCTC‐3′ for *CIPK24*, and 5′‐GCCTCTAGAATGGAGAGGCCAAGAGCCCCT‐3′ and 5′‐ATCCCGGGGAAGAGGTCAATGTCCAAGTCCG‐3′ for *PHOT2*. The amplified DNA fragments were cloned into the binary vectors pSPYNE‐35S and pSPYCE‐35S (Walter *et al*., 2004) via *Xba*I and *Sma*I sites. The pSPYNE‐35S and pSPYCE‐35S vectors bearing *PHOT1* cDNA were used as reported previously (Kaiserli *et al*., 2009). *Agrobacterium tumefaciens* (GV3101) was transformed with the resulting plasmid vectors and used for transformation of *N. benthamiana*. Agrobacteria‐mediated co‐infiltration of *N. benthamiana* leaves with pSPYNE and pSPYCE containing the indicated inserts was performed as previously described (Walter *et al*., 2004; Kaiserli *et al*., 2009). Detection of reconstituted YFP fluorescence was monitored 2.5 days post‐infiltration using a confocal microscope (Zeiss LSM510 and Leica SP8) (Walter *et al*., 2004; Kaiserli *et al*., 2009). Total YFP fluorescence from seven separate images and three independent experiments was quantified using Fiji (ImageJ) (Schindelin *et al*., 2012).

### Expression of *CIPK23*

RT‐PCR was performed as described previously (Inoue *et al*., 2008a). GCPs and MCPs were enzymatically prepared from rosette leaves as reported by Ueno *et al*. (2005) with slight modifications. Total RNAs were extracted from GCPs, MCPs, rosette leaves, petioles, inflorescence stems, and roots of 4‐week‐old wild‐type (Col) plants or from the aerial parts of *cipk23‐1* and *cipk23‐5* mutants with ISOGEN (Nippon Gene). First‐strand cDNAs were synthesized from 2 µg of each total RNA by SuperScript III reverse transcriptase using oligo(dT)_12‐18_ primer (Invitrogen). Full‐length *CIPK23* cDNAs were amplified by PCR using the following oligonucleotide primers: 5′‐ATGGCTTCTCGAACAACGCCTTCAC‐3′ and 5′‐TTATGTCGACTGTTTTGCAATTGTCCG‐3′ (Figure S3b). For amplification of the fragment of *CIPK23* cDNA, PCR was performed using the following oligonucleotide primers: 5′‐AGTTTCAAACTGCTTCTGCTCCAC‐3′ and 5′‐ACGAGGATTACATTTGCTGAGGTC‐3′ (Figure S3c). As an internal standard, a fragment of *ACT8* was used with the primers 5′‐ACTTTACGCCAGTGGTCGTACAAC‐3′ and 5′‐AAGGACTTCTGGGCACCTGAATCT‐3′.

### Measurement of stomatal aperture

Stomatal aperture in the leaf abaxial epidermis was measured according to previous methods (Inoue *et al*., 2008b; de Carbonnel *et al*., 2010) with a modification. To determine the stomatal opening, the epidermal fragments were incubated in 2 ml buffer containing 5 mm MES/bistrispropane (pH 6.5), 50 mm KCl, and 0.1 mm CaCl_2_, and illuminated with light for 3 h at room temperature (Figure 4a). To determine the stomatal opening by FC, the epidermis were incubated in 2 ml of buffer containing 5 mm MES/bistrispropane (pH 6.5), 10 mm KCl, 0.1 mm CaCl_2_, and FC at indicated concentrations for 3 h (Figure 5; Figure S5).

### Phenotypic analyses of phototropin‐mediated responses

Phototropic curvature of etiolated seedlings, chloroplast relocations and leaf flattening of rosette leaves, and stomatal conductance in intact leaves were determined according to previous methods (Doi *et al*., 2004; Inoue *et al*., 2008a, 2008b, 2011, 2017; Takemiya *et al*., 2013).

### H^+^ pumping and H^+^‐ATPase phosphorylation in guard cell protoplasts

GCPs were enzymatically prepared from rosette leaves and used for the measurements of H^+^ pumping, immunoblotting, and far‐Western blotting according to previously described methods (Ueno *et al*., 2005; Inoue *et al*., 2008b; Takemiya *et al*., 2013). Immunoblotting and far‐Western blotting were performed using anti‐H^+^‐ATPase antibodies and GST‐14‐3‐3 protein (GF14phi) to determine the amount of H^+^‐ATPase and the levels of H^+^‐ATPase phosphorylation, respectively.

### Isolation of guard cell protoplasts and whole‐cell K^+^ current recordings

GCPs for whole‐cell K^+^ current recordings were isolated as described previously (Zhao *et al*., 2012). Prior to each experiment, epidermal peels were obtained carefully from the abaxial surface of the youngest and fully expanded leaves of 2‐week‐old Arabidopsis and cut into pieces of 5 mm length. The epidermal strips were exposed to enzyme buffer (1.3% cellulase RS, 0.0075% pectolyase Y‐23, 0.25% BSA, 0.5 mm ascorbate (pH 5.5), and osmolality at 460 mOmol kg^−1^ adjusted with sorbitol) for approximately 20 min.

Whole‐cell K^+^ current recordings patch‐clamp pipettes were pulled with a vertical puller (model PC‐10; Narishige), modified for two‐stage pulls, and fire‐polished by a microforge (model MF‐90; Narishige) before using. The pipette solution typically contained 100 mm K‐glutamate, 2 mm MgCl_2_, 1.1 mm Mg‐ATP, 10 mm Hepes (4‐[2‐hydroxyethyl]‐1‐piperazineethanesulfonic acid)‐KOH (pH 7.2). Osmolality of the pipette solution was adjusted at 510 mOsmol kg^−1^ with d‐sorbitol. Bath solutions contained 10 mm K‐glutamate, 2 mm MgCl_2_, 0.5 mm CaCl_2_, and 10 mm Mes‐KOH (pH 5.5), and the osmolarity was adjusted to 460 mOsmol kg^−1^ with d‐sorbitol. Whole‐cell currents were measured in response to 3‐sec voltage pulses from −190 to −10 mV in 20 mV steps for the K^+^_in_ channel currents, using an EPC‐9 patch‐clamp amplifier (HEKA Elektronik, Lambrecht, Germany).

At the onset of each patch‐clamp experiment, the room was illuminated with a dim red light (0.2 µmol m^−2^ sec^−1^). After the whole‐cell configuration was obtained, the membrane was clamped to −52 mV (holding potential). When the recorded currents appeared stable, the patch‐clamped cells were illuminated with 100 µmol m^−2^ sec^−1^ blue light for 30 sec. A fiber optic halogen light source (Nikon, Tokyo) and Plexiglas filters were used for light treatments. Whole‐cell data were low‐pass filtered with a cutoff frequency of 2.9 kHz and analyzed with the software PLUSEFIT (version 8.7) and IGOR 3.0. The final whole‐cell currents were expressed in pA (Figure S6; Yin *et al*., 2013) or as currents per unit capacitance (pA pF^−1^) (Figure 6b–d) to account for variations in the cell surface area. Data are presented as means ± SE.

### Thio‐phosphorylation in *in vitro* phosphorylation assay

Detection of thio‐phosphorylation derived from phot1 kinase activity was performed as previously described (Schnabel *et al*., 2018). The cDNA fragments of *GST‐PHOT1‐T740G* and *GST‐CIPK23* were cloned into the pSP64 vector. GST‐phot1_T740G_ and GST‐CIPK23 were expressed using the TNT SP6 High‐Yield Wheat Germ Protein Expression System (Promega), with 2 µg of each vector for a 20 µl reaction, and 10 µm flavin mononucleotide as a chromophore. Protein expression was performed at room temperature in the dark for 2 h. The *in vitro* kinase assay was performed as previously described (Sakai *et al*., 2001) with modifications. Ten µl of *in vitro* protein expression extract and *N^6^*‐benzyl‐ATPγS (500 µm) were mixed in each reaction volume (20 µl) with phosphorylation buffer (37.5 mm Tris‐HCl (pH 7.5), 5.3 mm MgSO_4_, 150 mm NaCl, and 1 mm EGTA). Light was irradiated to the samples for 20 sec at a total fluence of 60 000 µmol m^−2^. Reactions were incubated in the dark at room temperature for 5 min and terminated by adding EDTA (pH 8.0) to a final concentration of 20 mm. Thio‐phosphorylated molecules were alkylated by adding *p*‐nitrobenzyl mesylate at a final concentration of 2.5 mm and incubated for 2 h. Then, protein samples were subjected to SDS‐PAGE and Western blotting as described previously (Schnabel *et al*., 2018). Protein thio‐phosphorylation and GST protein were detected using a rabbit anti‐thiophosphoester monoclonal antibody and a goat anti‐GST monoclonal antibody, respectively, as primary antibodies. HRP‐conjugated anti‐rabbit or anti‐goat secondary antibody and Pierce ECL Plus Western blotting substrate (Thermo Fisher Scientific) were used to develop the signals.

## ACCESSION NUMBERS

*PHOT1* (AT3G45780), *PHOT2* (AT5G58140), *CIPK23* (AT1G30270), *BLUS1* (AT4G14480), *CIPK1* (AT3G17510), *CIPK24* (AT5G35410), *ACT8* (AT1G49240).

## CONFLICT OF INTEREST

The authors of the manuscript declare no conflict of interest.

## AUTHOR CONTRIBUTIONS

SI and KS conceived the original screening and research plans; SI, MS, KS, YE, TS, TK, JMC, XZ, and KS designed and supervised the experiments; SI, EK, XZ, TW, and AT performed experiments; SI, EK, MO, HT, TS, XZ, JMC, and KS analyzed the data; SI, EK, TW, XZ, JMC, and KS wrote the article.

## Supporting information


**Figure S1.** Representative confocal images of reconstitution of YFP fluorescence upon interaction between phot1 and CIPK23 and corresponding negative controls.

**Figure S2.** BiFC analysis showing the interactions between phototropins and CIPK24 and CIPK24 homodimerization in *Nicotiana benthamiana* leaf epidermal cells.

**Figure S3.** Transfer DNA insertional mutants used in this study and expression of *CIPK23* in various tissues.

**Figure S4.** Blue light‐induced activation of the plasma membrane H^+^‐ATPase in GCPs from wild‐type (Col) and *cipk23‐5* plants.

**Figure S5.** Stomatal opening in response to fusicoccin in darkness.

**Figure S6.** Effects of blue light on whole‐cell inward‐rectifying K^+^ channel currents in wild‐type (Col), *cipk23‐5*, and *blus1‐3* GCPs.

**Figure S7.** CIPK23 is not phosphorylated by phot1 in the *in vitro* kinase assay.

**Figure S8.** Phylogenetic relationships among *Arabidopsis thaliana* CIPK family members.

**Figure S9.** Gene expression levels of *CIPK23*, *CIPK3*, *CIPK9*, *CIPK26*, *CIPK6*, and *CIPK16* in guard cells.

**Figure S10.** Proposed blue light signalings in guard cells.

**Table S1.** Blue light‐dependent H^+^‐pumping in GCPs from wild‐type (Col) and *cipk23‐5* plants.

## Data Availability

All relevant data are included in the manuscript and its supporting materials.
