# Supplementary figures and images for: CIPK23 regulates blue light‐dependent stomatal opening in Arabidopsis thaliana
Source: Plant J. 2020 Sep 1;104(3):679–92. doi: 10.1111/tpj.14955 (PMC7693358; doi:10.1111/tpj.14955)

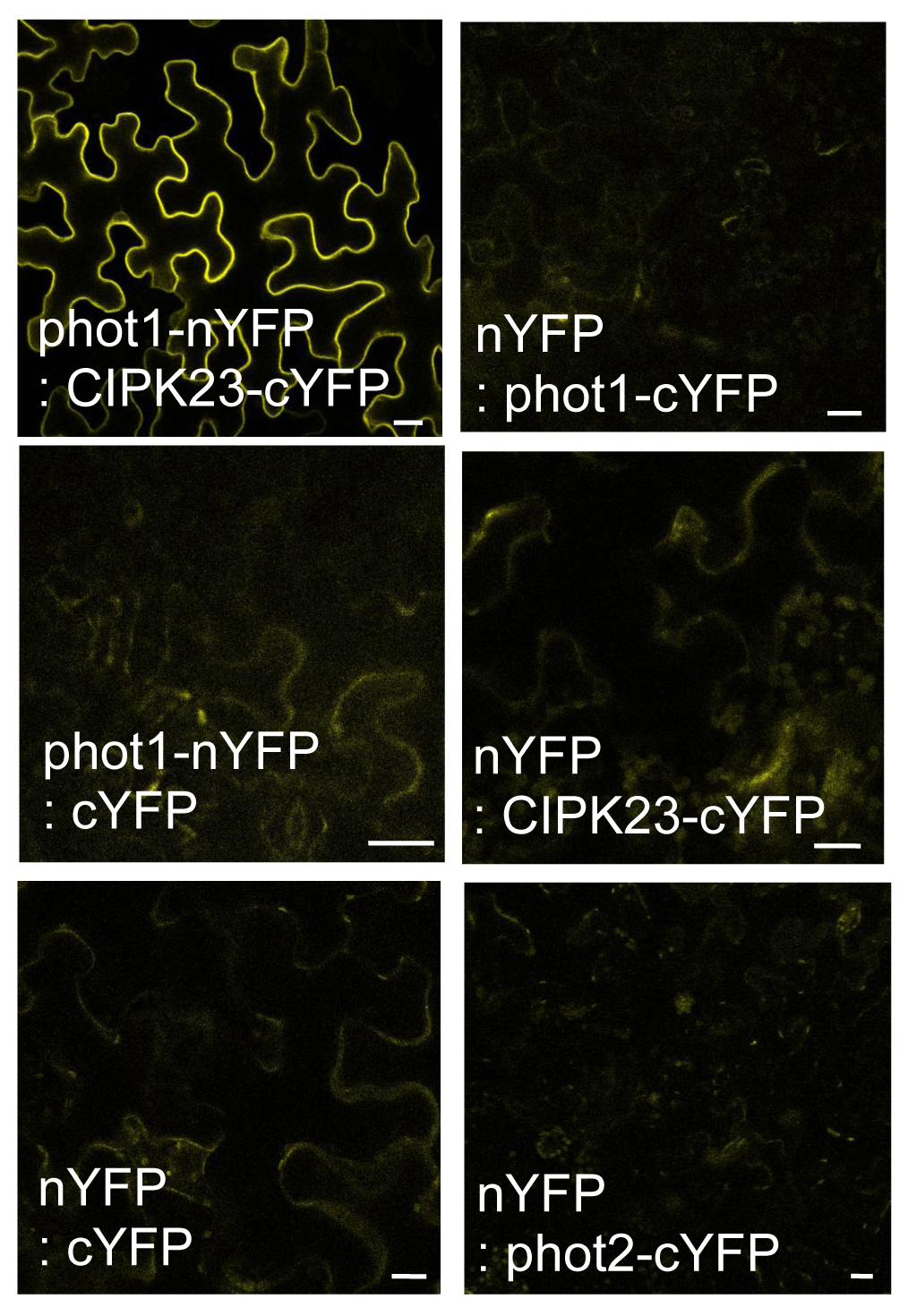

Supplement: Supplementary file 1 — Figure S1. Representative confocal images of reconstitution of YFP fluorescence upon interaction between phot1 and CIPK23 and corresponding negative controls. [file TPJ-104-679-s001.tif]

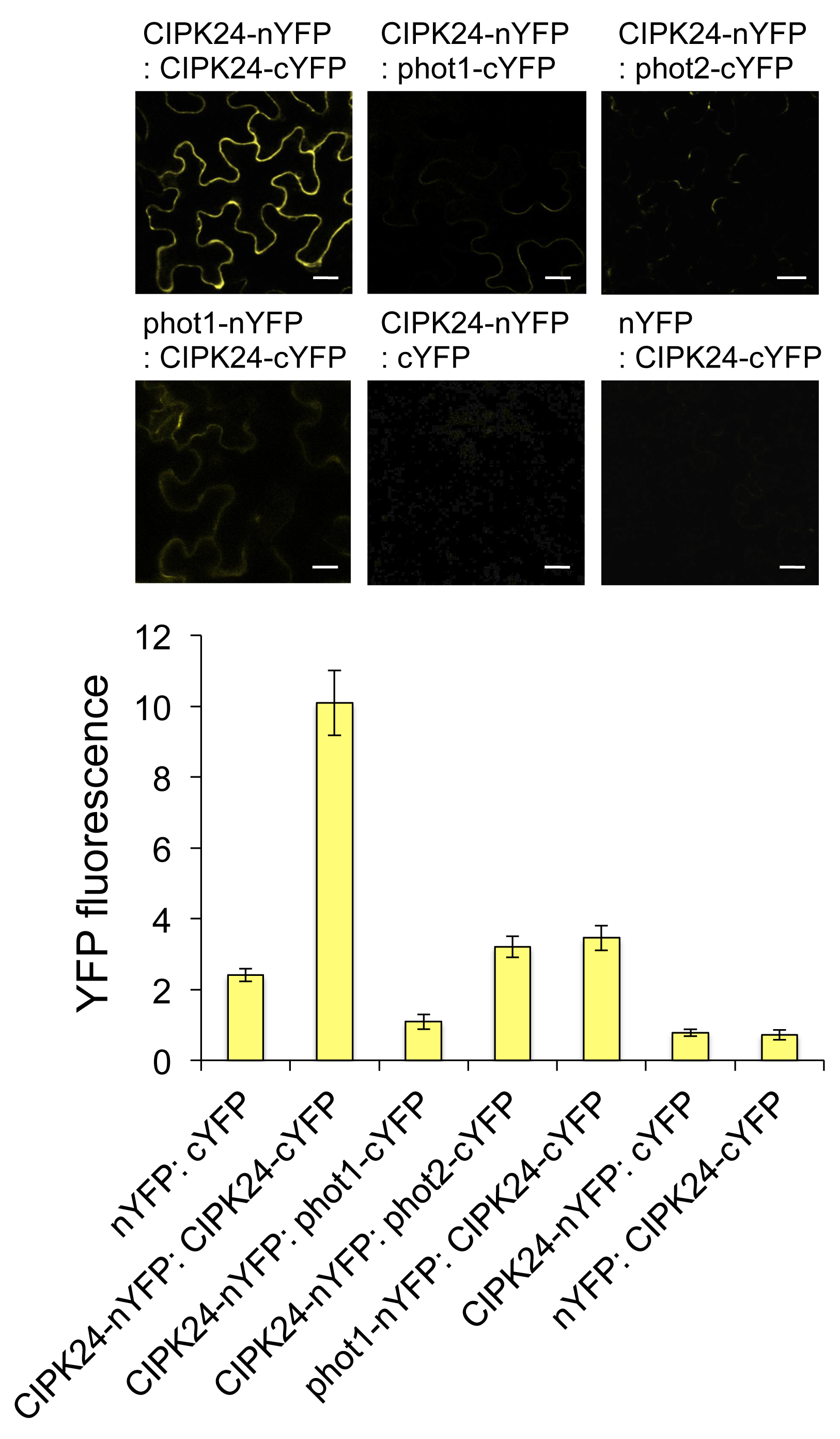

Supplement: Supplementary file 2 — Figure S2. BiFC analysis showing the interactions between phototropins and CIPK24 and CIPK24 homodimerization in Nicotiana benthamiana leaf epidermal cells. [file TPJ-104-679-s002.tif]

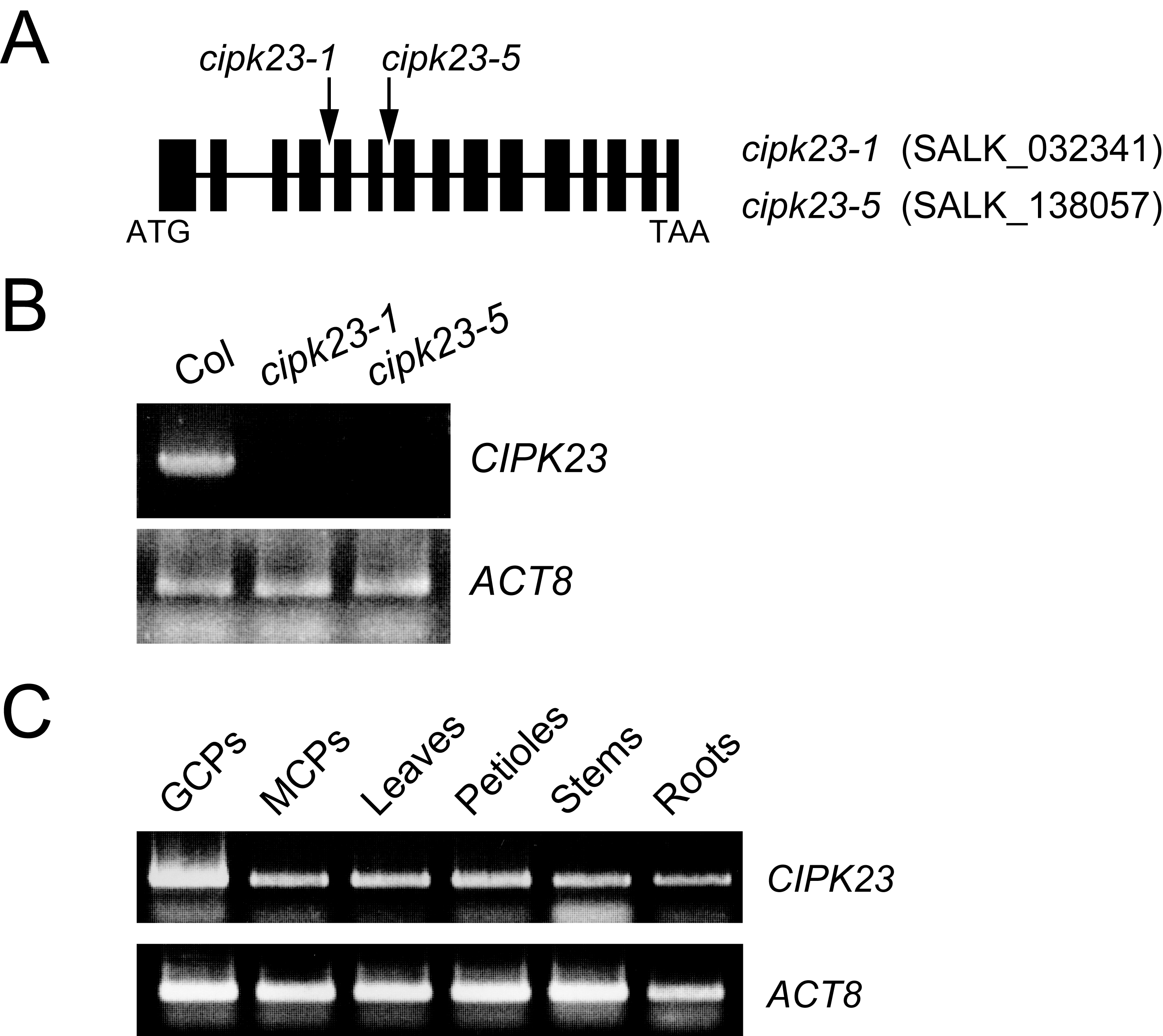

Supplement: Supplementary file 3 — Figure S3. Transfer DNA insertional mutants used in this study and expression of CIPK23 in various tissues. [file TPJ-104-679-s003.tif]

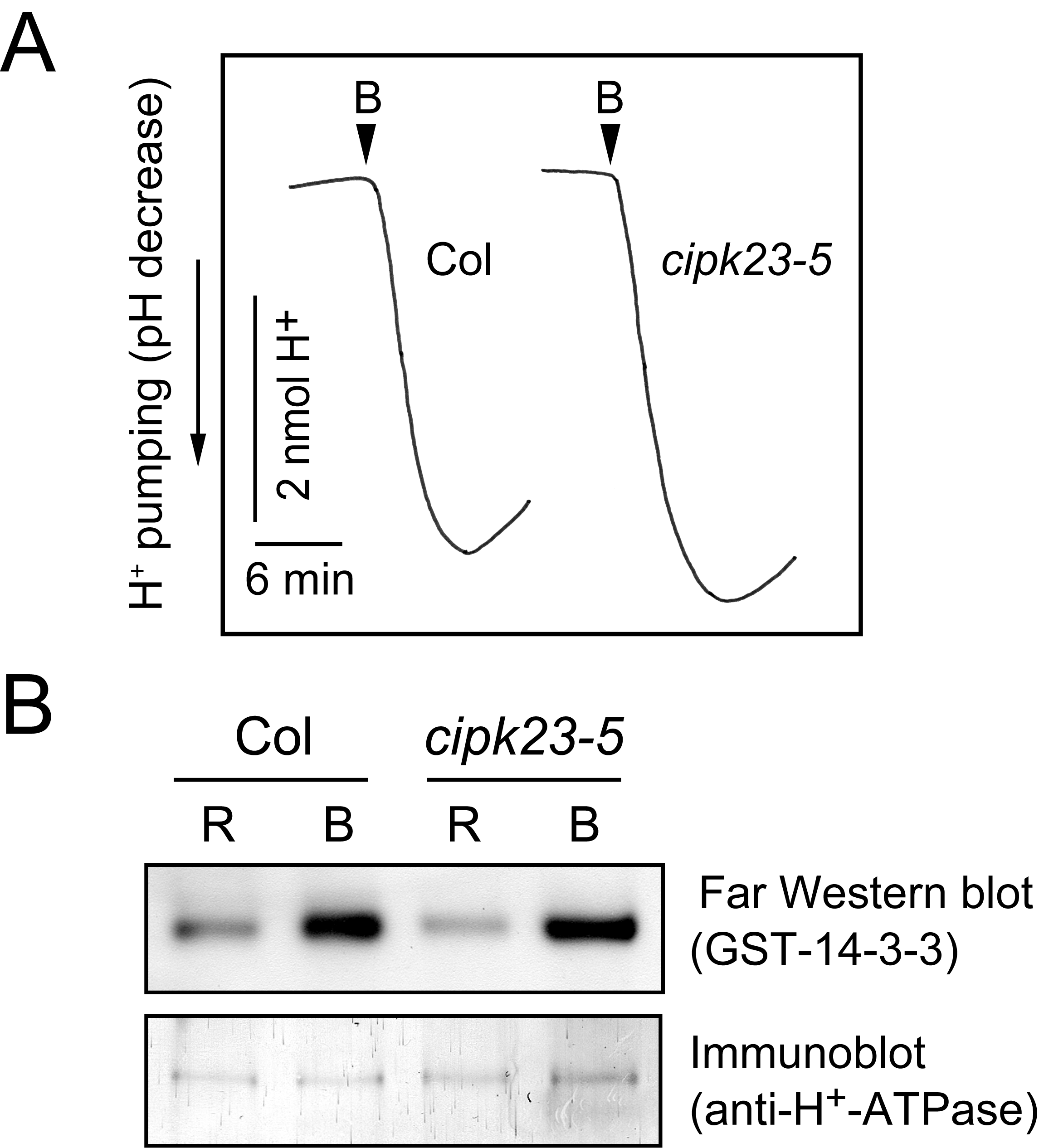

Supplement: Supplementary file 4 — Figure S4. Blue light‐induced activation of the plasma membrane H+‐ATPase in GCPs from wild‐type (Col) and cipk23‐5 plants. [file TPJ-104-679-s004.tif]

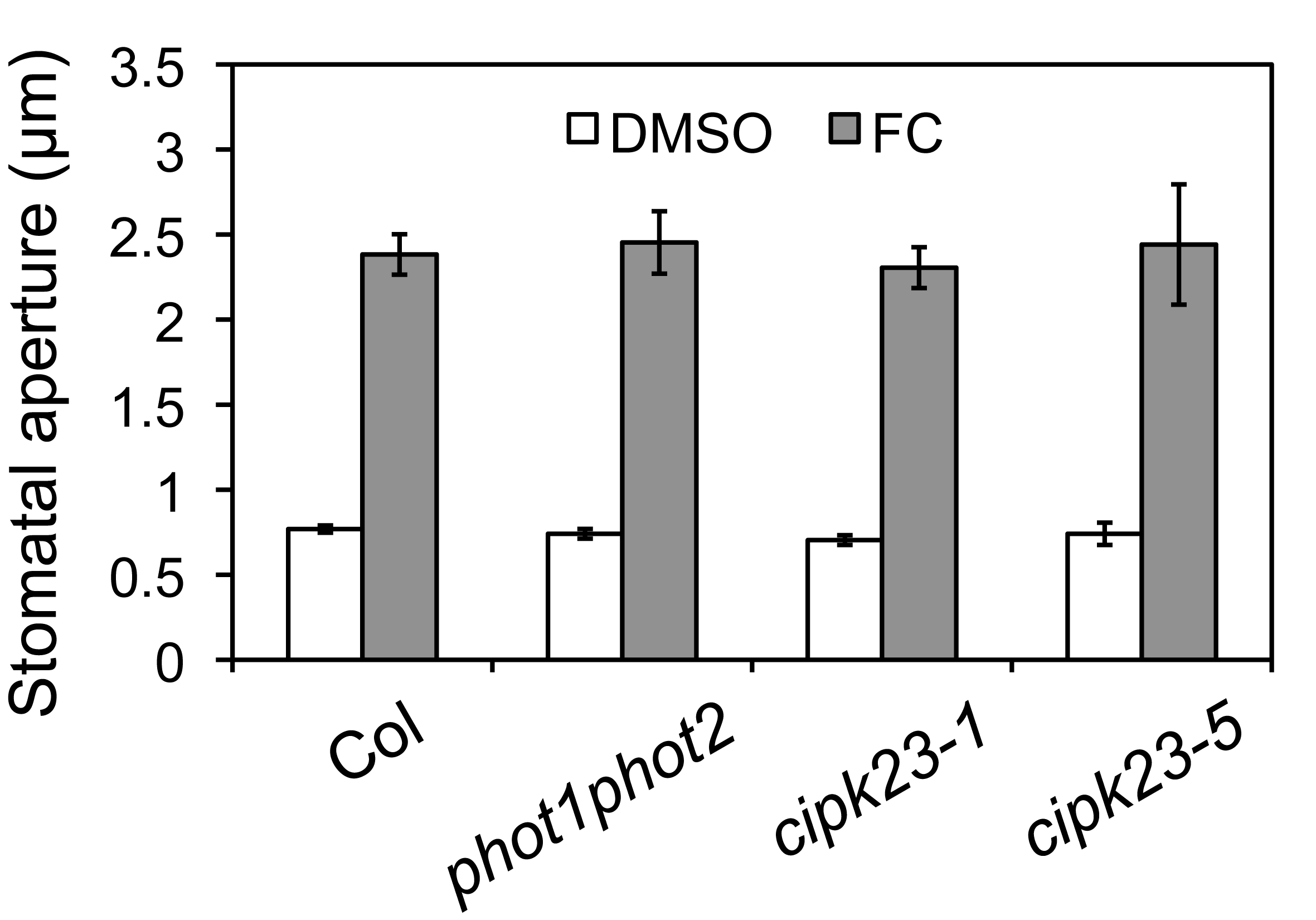

Supplement: Supplementary file 5 — Figure S5. Stomatal opening in response to fusicoccin in darkness. [file TPJ-104-679-s005.tif]

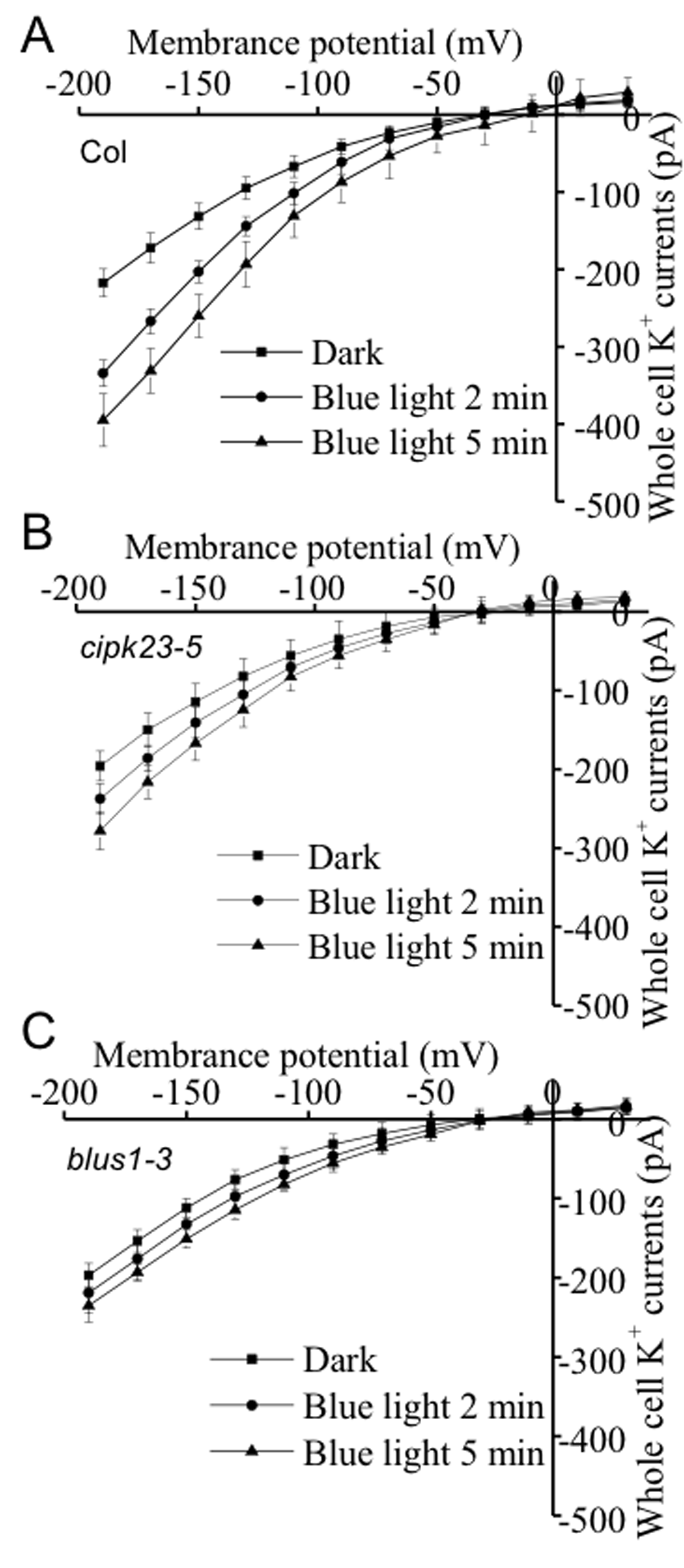

Supplement: Supplementary file 6 — Figure S6. Effects of blue light on whole‐cell inward‐rectifying K+ channel currents in wild‐type (Col), cipk23‐5, and blus1‐3 GCPs. [file TPJ-104-679-s006.tif]

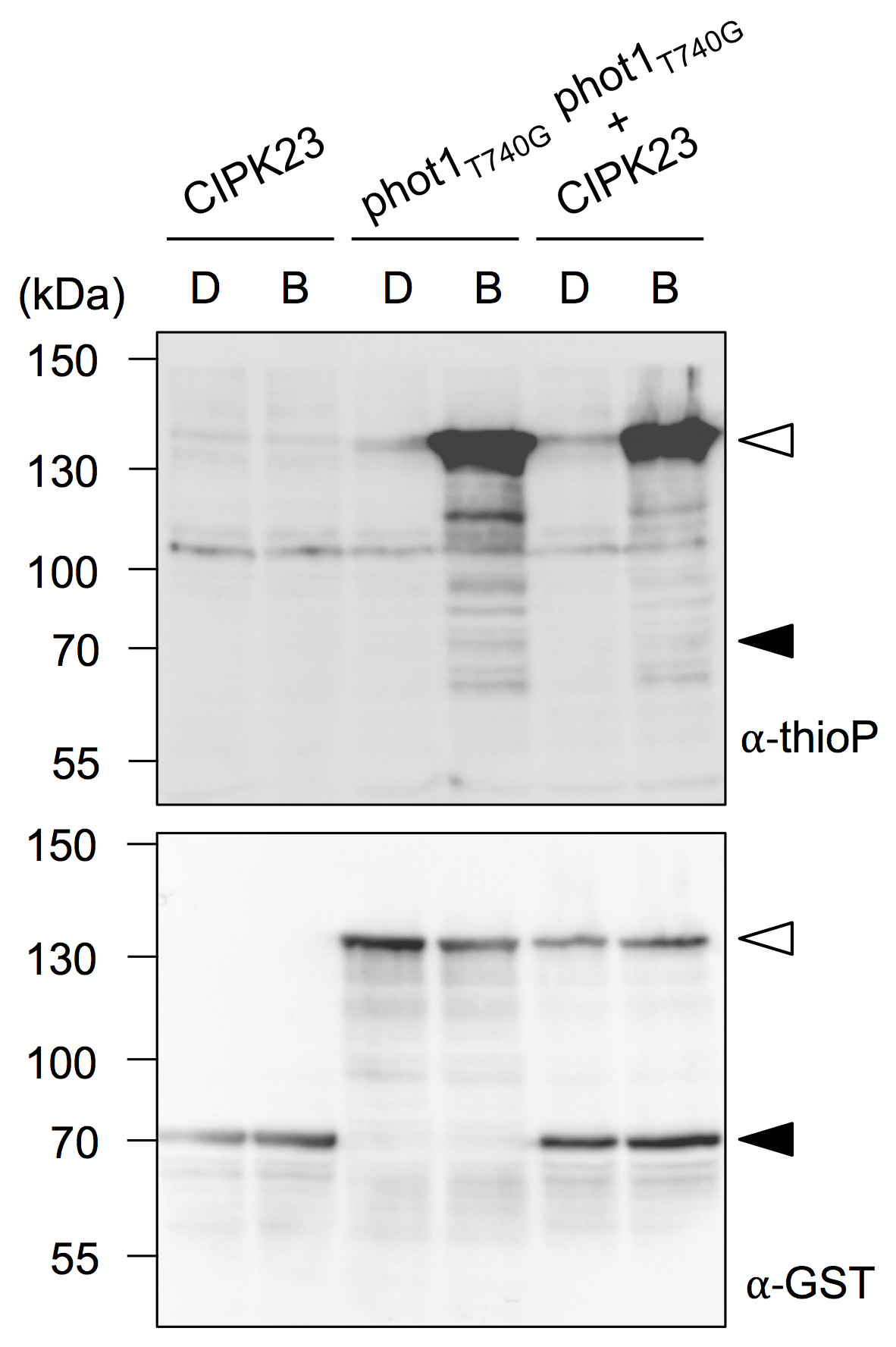

Supplement: Supplementary file 7 — Figure S7. CIPK23 is not phosphorylated by phot1 in the in vitro kinase assay. [file TPJ-104-679-s007.tiff]

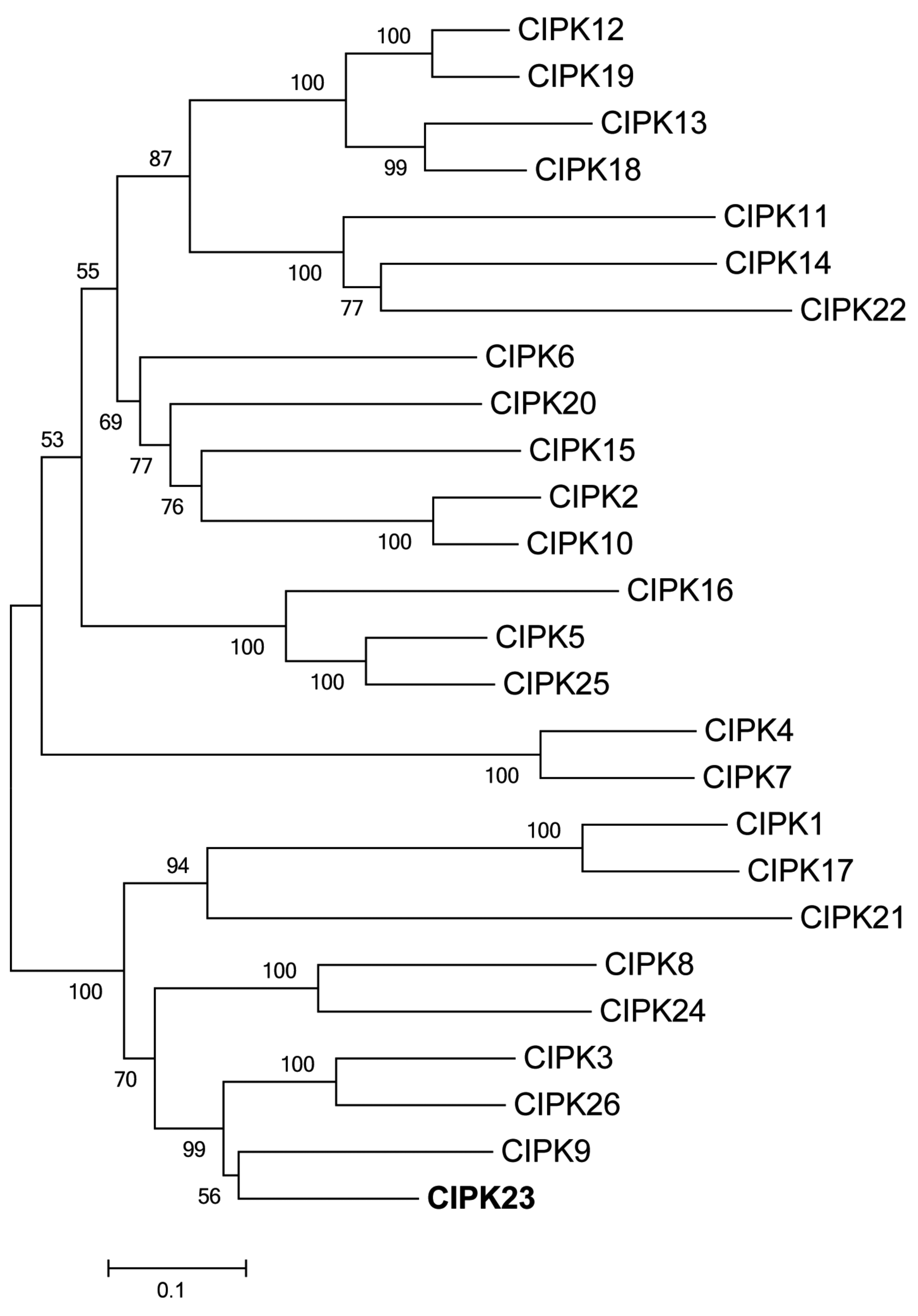

Supplement: Supplementary file 8 — Figure S8. Phylogenetic relationships among Arabidopsis thaliana CIPK family members. [file TPJ-104-679-s008.tif]

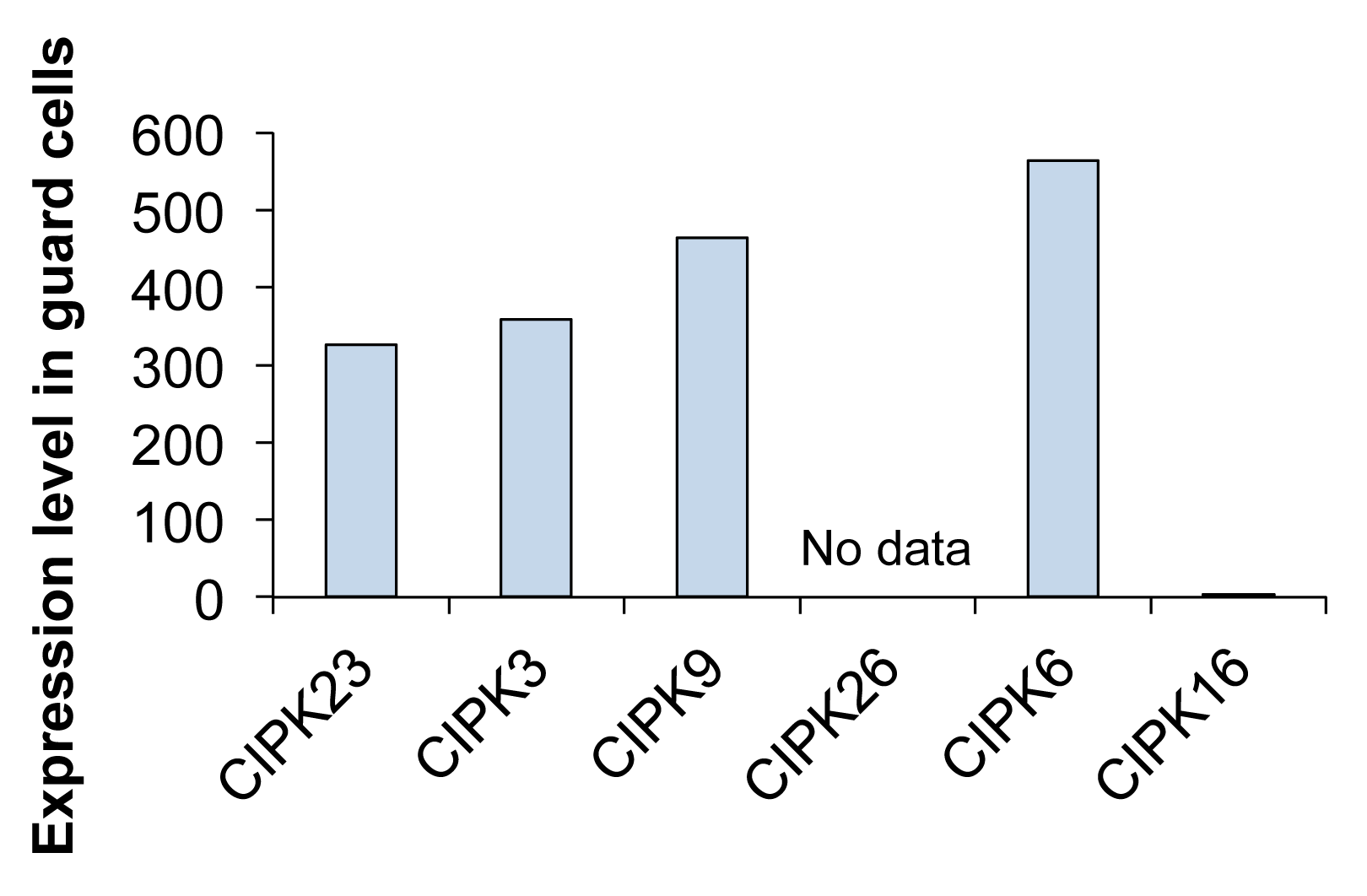

Supplement: Supplementary file 9 — Figure S9. Gene expression levels of CIPK23, CIPK3, CIPK9, CIPK26, CIPK6, and CIPK16 in guard cells. [file TPJ-104-679-s009.tif]

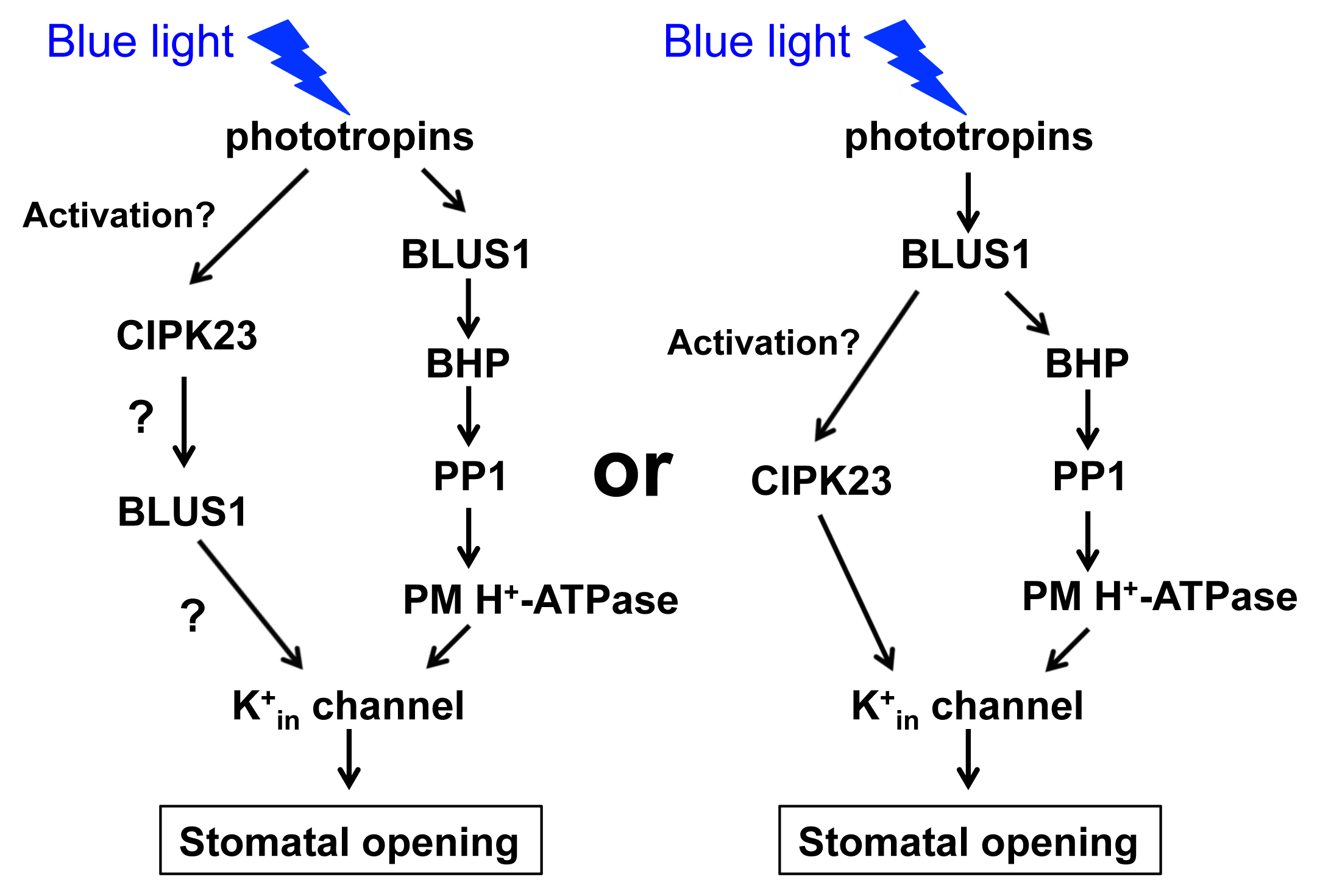

Supplement: Supplementary file 10 — Figure S10. Proposed blue light signalings in guard cells. [file TPJ-104-679-s010.tif]
