# Supplementary material for: CIPK23 regulates blue light‐dependent stomatal opening in Arabidopsis thaliana
Source: Plant J. 2020 Sep 1;104(3):679–92. doi: 10.1111/tpj.14955 (PMC7693358; doi:10.1111/tpj.14955)
Supplement: Supplementary file 11 — Table S1. Blue light‐dependent H+‐pumping in GCPs from wild‐type (Col) and cipk23‐5 plants. [file TPJ-104-679-s011.docx]

**Table S1. Blue light-dependent H^+^-pumping in GCPs from Col and *cipk23-5.***

|  | Col | *cipk23-5* |
| --- | --- | --- |
| Maximum rate (nmol H^+^ h^-1^ µg protein^-1^) | 1.10±0.14 | 1.12±0.20 |
| Magnitude (nmol H^+^ µg protein^-1^) | 0.062±0.009 | 0.064±0.013 |

Experiments were performed as described in Figure S4. The maximum rate was measured 2.0 min after the start of blue light-pulse. Data represent the means ±SE of three independent experiments.
